# Triple‐Shell Hollow FeS/MoS_2_ Heterostructure Anodes: Synergistic Effects of Built‐In Electric Field on Ultra‐Stable Sodium Storage

**DOI:** 10.1002/advs.202509997

**Published:** 2025-08-26

**Authors:** Mingyang Chen, Shaonan Gu, Junhui Li, Yuxin Dai, Yanyan He, Bin Sun, Tingting Gao, Liqiang Xu, Guowei Zhou

**Affiliations:** ^1^ Key Laboratory of Fine Chemicals in Universities of Shandong Jinan Engineering Laboratory for Multi‐scale Functional Materials School of Chemistry and Chemical Engineering Qilu University of Technology (Shandong Academy of Sciences) Jinan 250353 P. R. China; ^2^ Key Laboratory of Colloid and Interface Chemistry School of Chemistry and Chemical Engineering Shandong University Jinan 250100 P. R. China

**Keywords:** built‐in electric field, heterostructure, hollow sphere anode, sodium‐ion batteries, tripe‐shell sulfides

## Abstract

Metal sulfides are intensively pursed as promising anode materials for sodium‐ion batteries (SIBs) owing to their high theoretical capacities, abundant and inexpensive raw materials, however, challenges remain in designing their structures, particularly due to the slow Na⁺ storage kinetics in individual sulfide, and unshaped and inefficient heterostructure persists the issue of low intrinsic ion conductivity. Herein, hollow triple‐shell FeS/MoS_2_@NC structure by integrating molecular and microstructural engineering is constructed. The intimate connection between FeS and MoS_2_ in FeS/MoS_2_@NC arises from the simultaneous sulfidation of Fe_2_(MoO_4_)_3_. This hollow multi‐shell structure, along with the high effective built‐in electric field between the sulfides, effectively mitigates the volume expansion and propelled Na⁺ storage kinetics. Additionally, superparamagnetic Fe0 nanodots appeared from FeS reduction at low voltage during charge–discharge cycles facilitate Na+ storage. As a result, the hollow triple‐shell FeS/MoS_2_@NC presented high reversible capacity (611.8 mAh g^−1^ at 0.1 A g^−1^) and ultra‐stable cycling span‐life (451.5 mAh g^−1^ after 9000 cycles at 5 A g−^1^). In addition, the assembled Na_3_V_2_(PO_4_)_3_@rGO//FeS/MoS_2_@NC coin‐type full cell exhibited remarkable electrochemical performance (322.2 mAh g^−1^ after 400 cycles at 1 A g^−1^). This work will stimulate the further development of metal sulfide heterostructure and also provide new perspectives on high performance SIBs anodes.

## Graphical abstract

MoS_2_ as anode material for SIBs usually suffers from slow Na⁺ diffusion and low conductivity. In this work, triple‐shell FeS/MoS_2_@NC structured material as anode are designed, where the FeS/MoS_2_ heterostructure interface introduced internal electric field and spin‐polarized surface capacitance could boost Na⁺ diffusion dynamics and further enhance Na⁺ storage, ensuring excellent long‐cycle stability (451.5 mAh g^−1^ for 9 000 cycles).

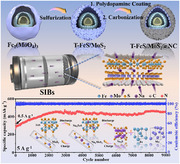

## 1 Introduction

Sodium ion batteries (SIBs) have attracted promising attention in recent years due to their commercial cost and abundant natural resources, as well as their higher safety compared with lithium ion batteries.^[^ ^1^, ^2^, ^3^ ^]^ Unfortunately, current graphite anodes are not ideal for SIBs due to the larger radius of the Na^+^ (≈0.102 nm).^[^ ^4^, ^5^, ^6^ ^]^ The large ionic radius also causes delayed Na^+^ transportation, which has a significant impact on the electrochemical performance of the anodes. The key to achieving high performance anodes of SIBs lies in effective fast Na^+^ transport kinetics in long cycles and mitigated volume expansion during Na^+^ insertion and desertion by rational component and structural design.^[^ ^7^, ^8^, ^9^ ^]^

Transition metal sulfides possess unique advantages as anode materials for SIBs, including their high theoretical capacities and favorable electrochemical properties.^[^ ^10^, ^11^ ^]^ The presence of transition metal sulfides, such as Fe_1–x_S,^[^ ^12^ ^]^ WS_2–x_,^[^ ^13^ ^]^ MoS_2_@Mxene,^[^ ^14^ ^]^ etc., introduces a range of redox‐active sites that can promote faster Na⁺ diffusion, further contributing to their high‐rate capability and long cycling stability. Moreover, heterostructures based on transition metal sulfides, such as Fe_1–x_S@Sb@C,^[^ ^15^ ^]^ CoS/MoS_2_,^[^ ^16^ ^]^ MoS_2_/V_2_O_3_@C‐rGO,^[^ ^17^ ^]^ can significantly improve Na^+^ storage kinetics by creating built‐in electric field at phase interfaces, thereby reducing ion diffusion barriers and promoting faster charge transfer. Among these transition metal sulfides, MoS_2_ is a promising candidate because of its structural similarity to graphite and the larger layer spacing (≈0.62 nm) promoting reversible insertion/desertion of Na^+^ with better cycling stability.^[^ ^18^, ^19^, ^20^, ^21^ ^]^ However, the semiconductor nature of 2H‐MoS_2_ leads to a different rate of Na^+^ storage reaction between the edge and the center of MoS_2_, resulting in an impeded Na^+^ diffusion and reaction kinetics, which further limits the rate performance and cycling stability of the MoS_2_ anode.^[^ ^22^ ^]^ To address above issues of MoS_2_ as SIBs anode, coupled with another compounds into composites is an effective approach benefit from built‐in electric field between the phase interface of composite for facilitating the transportation of Na^+^ and electrons.^[^ ^23^ ^]^

Most of this complicated structural design has so far been done by growing a kind of metal sulfide on top of the other sulfide to form a heterogeneous structure. However, the unshaped and inefficient heterostructure means that the lower intrinsic ion conductivity remains largely unresolved. Meanwhile, the functional mechanism of varies component on heterostructure is not clear and rarely studied since the sulfides undergo oxidation and reduction during battery cycling. Therefore, it is imperative to comprehensive understand the influence of heterostructure on effective sodium storage at atomic level. In addition to fabricate heterostructures, rational nanostructure design has also been shown to be beneficial in improving the long cycle stability and reaction kinetics of anode.^[^ ^24^, ^25^, ^26^ ^]^ As an anode material for SIBs with high cycling stability, hollow multi‐shell structures (HoMS) with outer shells and internal cavities other than mitigate the volume changes and the structural stress brought by Na^+^ insertion and desertion, but can also ensure a larger effective electrode‐electrolyte contact and more active sites.^[^ ^27^ ^]^ As well, the parallel circuit formed by multiple shell layers can reduce the transfer resistance, thus enabling fast electrochemical reactions. Therefore, combining HoMS structural design with heterostructure fabrication is expected to obtain significantly improving cycling stability and Na^+^ storage kinetics.

In this work, FeS/MoS_2_@NC with triple‐shell structure encapsulated by nitrogen‐doped carbon (NC) derived from polydopamine (PDA) pyrolysis is achieved using carbonaceous microspheres as templates by a modified sequential template approach, in where the intimately contacted FeS/MoS_2_ heterostructure was born in situ from the sulfidation of Fe_2_(MoO_4_)_3_. Meanwhile, the heterostructure formed simultaneously by the bimetallic oxides greatly increases the contact area of FeS and MoS_2_ and exposes more active sites within an effective built‐in electric field. Furthermore, the conversion of FeS in the heterostructure into Fe nanodots (≈ 3 nm) at low voltage (below 0.3 V) facilitates Na^+^ storage, which is probably derived from the change of the spin state of Fe to high‐spin paramagnetic. In addition, the unique triple‐shell structure shortens the charge diffusion path and buffers the volume change during discharge and charge, resulting in cycling stability. Benefited from the above advantages, FeS/MoS_2_@NC exhibits impressive electrochemical performance with ultra‐long cycling stability, retaining a capacity of 451.5 mAh g^−1^ after 9000 cycles at a current density of 5 A g^−1^. The coin‐type full sodium‐ion battery (Na_3_V_2_(PO_4_)_3_@rGO//T‐FeS/MoS_2_@NC) showcased an impressive electrochemical performance, achieving 322.2 mAh g^−1^ after 400 cycles at a current density of 1 A g^−1^. For a fundamental understanding of the enhancement mechanism, the density functional theory calculations and in situ characterizations were further performed. This work sheds light on the atomic‐level mechanism and multi‐shell structure fabrication of the anode electrochemical processes and offers guidance for the design of sulfide‐based anodes for SIBs.

## 2 Results and Discussion

**Figure 1:**
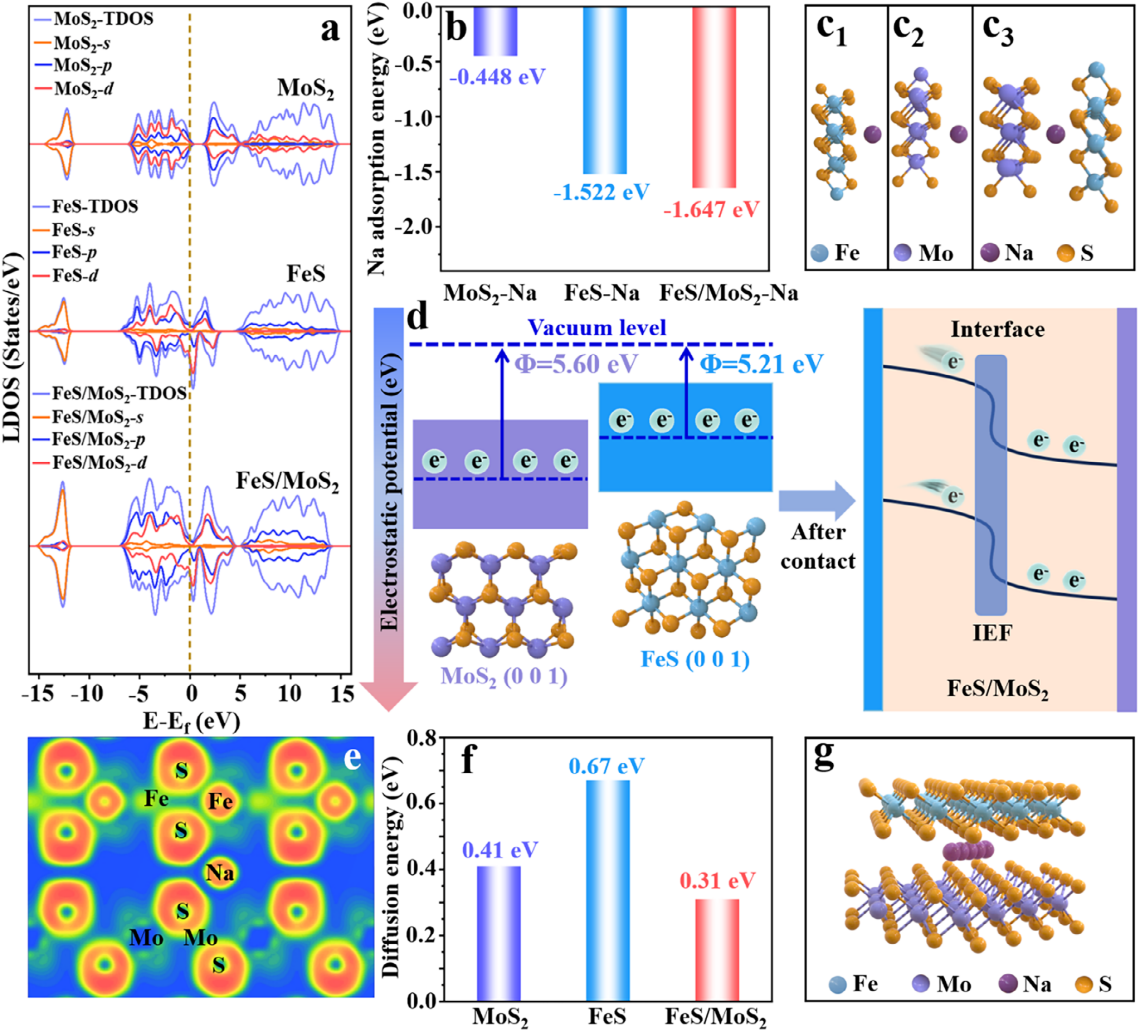
a) Local density of states (LDOS) of different calculated models. b) Na^+^ adsorption binding energies for different surfaces. (c_1_–c_3_) Schematic diagrams of Na adsorption structures on the surfaces of MoS_2_ (0 0 1), FeS (0 0 1), and FeS/MoS_2_. d) Schematic illustration of work function and built‐in electric field in FeS/MoS_2_ heterostructure. e) Electronic localization function (ELF) of Na adsorption in FeS/MoS_2_ heterostructure. f) Na^+^ diffusion energy barriers for MoS_2_, FeS, and FeS/MoS_2_. g) Optimized diffusion paths of Na^+^ in FeS/MoS_2_.

To significantly enhance the sodium storage performance of SIBs anode materials, FeS/MoS_2_@NC HoMS with an efficient heterostructure was synthesized in this work, aiming to explore the influence of the multi‐shell structure and heterostructure on the electrochemical performance of SIBs anodes. Theoretical DFT simulations were performed prior to experimentation to predict whether the structural and electronic properties of FeS/MoS_2_ could enhance Na^+^ storage.^[^ ^28^ ^]^ The local density of states (LDOS) and total density of states (TDOS) were implemented to reveal the conductivity of the theoretical model (**Figure** **1**a). The highest electronic state of FeS/MoS_2_ near the Fermi level indicates enhanced electron transfer efficiency during Na^+^ storage. The optimized configurations of Na^+^ absorbed on the surface of FeS (0 0 1), MoS_2_ (0 0 1), and FeS/MoS_2_ are displayed in Figure S1 (Supporting Information). Figure 1b,c present the Na^+^ adsorption binding energy and the side view of absorbed Na on the surface of FeS (0 0 1), MoS_2_ (0 0 1), and FeS/MoS_2_, respectively. The higher binding energy of FeS/MoS_2_ compared to the other models suggests that the heterostructure enhance the adsorption of Na^+^.^[^ ^29^ ^]^ The work function (*Φ*) was calculated to investigate the built‐in electric field in heterostructures (Figure S2, Supporting Information). The Fermi level of the FeS (0 0 1) model is significantly higher than that of the MoS_2_ (0 0 1) model, causing spontaneous electron flow from FeS to MoS_2_ at the interface, indicating that the direction of the built‐in electric field is from FeS to MoS_2_, as shown in Figure 1d. Figure 1e presents the electron localization function (ELF) for the adsorption side view of Na between FeS/MoS_2_, which evaluates the degree of charge localization around each atom in the structure. It is observed that S atoms near the Na atom show weaker electron localization, which facilitates the intercalation and deintercalation of Na^+^ in heterostructrue.^[^ ^30^ ^]^ Figure 1f,g presents the diffusion barriers for Na^+^ across the three models, along with a side view of the diffusion path of Na^+^ in FeS/MoS_2_ (refer to Figure S3, Supporting Information for the optimized configuration). As anticipated, the diffusion barrier of FeS/MoS_2_ is lower than that of FeS and MoS_2_, further demonstrating that the built‐in electric field in the heterostructure facilitates the migration of sodium ions.

**Figure 2:**
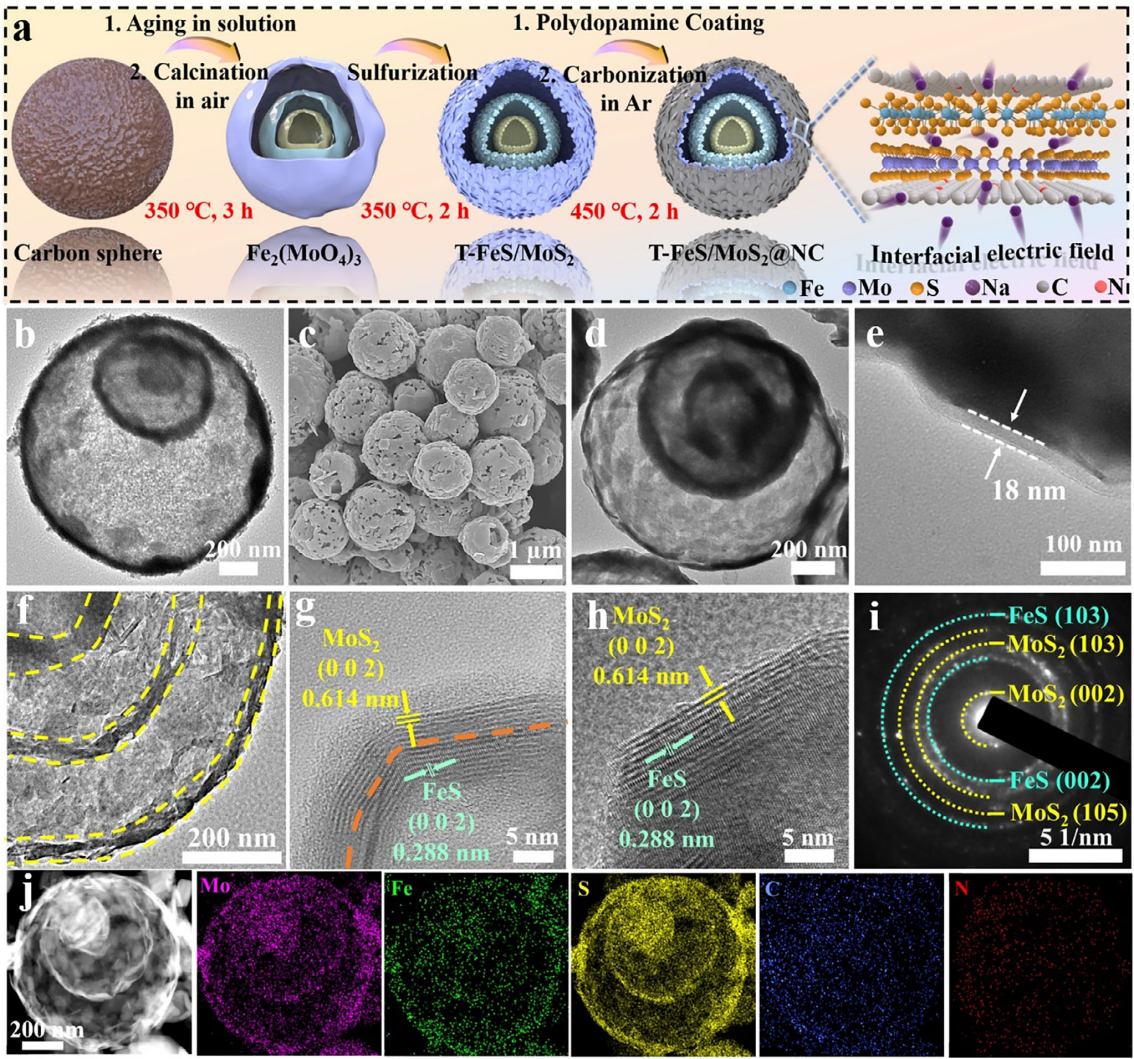
a) Schematic of the synthesis route for T‐FeS/MoS_2_@NC. b) TEM image of Fe_2_(MoO_4_)_3_ precursor. c) SEM image and d–f) TEM images of T‐FeS/MoS_2_@NC at various magnifications. g,h) HRTEM images of T‐FeS/MoS_2_@NC. i) SAED pattern and j) Element distribution of T‐FeS/MoS_2_@NC.

The aforementioned DFT calculations predict that the built‐in electric field in heterostructures enhances electron conductivity and promotes Na⁺ mass transfer, thereby improving the sodium‐ion storage performance. To further enhance the electrochemical performance, the triple‐shell FeS/MoS_2_@NC spheres (T‐FeS/MoS_2_@NC) was synthesized. The preparation process of T‐FeS/MoS_2_@NC is depicted in **Figure** **2**a. First, Fe_2_(MoO_4_)_3_ HoMS precursor was synthesized using a modified sequential template approach. Carbon microspheres were synthesized via a hydrothermal polymerization of sucrose solution approach. Fourier‐transform infrared (FTIR) spectroscopy (Figure S4, Supporting Information) results reveal that the characteristic absorption bands at 3400 and 1700 cm^−1^ correspond to the stretching vibrations of hydroxyl (–OH) and carbonyl (C═O) groups, respectively. These oxygen‐containing functional groups serve as active sites for subsequent coordination and anchoring of metal ions. Specifically, metal ions (Mo^6+^, Fe^3+^) infiltrate into the carbon sphere template during the aging process, and then a metal oxide layer forms on the surface of the carbon sphere upon subsequent calcination in air. As the metal oxide layer becomes sufficiently dense, its rigidity surpasses the adhesive force between the metal oxide and the carbon sphere surface, resulting in the formation of a shell structure, and the above process is repeated to obtain a triple‐shell Fe_2_(MoO_4_)_3_ precursor.^[^ ^31^ ^]^ Second, the T‐FeS/MoS_2_@NC composite is achieved by a vapor‐phase sulfurization process of Fe_2_(MoO_4_)_3_ precursor. Finally, the T‐FeS/MoS_2_@NC composite is coated with polydopamine, followed by calcination in an argon atmosphere to yield the T‐FeS/MoS_2_@NC spherical structure.

Amorphous carbon microspheres served as a sacrificial template (Figure S5a), and EDS elemental mapping confirmed that the carbon spheres were uniformly coated with Fe, Mo elements (Figure S5b–d). During the heating treatment, carbon spheres began to shrink at ≈ 300 °C, with the formation of the Fe_2_(MoO_4_)_3_ shell layer occurring at 450 °C (Figure S6a,b, Supporting Information). The XRD pattern of the Fe_2_(MoO_4_)_3_ precursor is presented in Figure S7 (Supporting Information). SEM and TEM images demonstrated that the Fe_2_(MoO_4_)_3_ precursor has a particle size ranging from 800 to 2000 nm (Figure S8, Supporting Information) with an internal uniform triple‐shell structure (Figure 2b), while the large internal voids were generated by carbon decomposition during calcination, consistent with the significant weight loss of 64.5% (Figure S9, Supporting Information). The structure of FeS/MoS_2_ retained the triple‐shell spherical structure of Fe_2_(MoO_4_)_3_ precursor, which is clearly visible in the SEM and TEM images (Figures S10 and S11, Supporting Information). The detailed structural characterization of T‐FeS/MoS_2_@NC is shown in Figure 2c–j. The SEM image displays that the uniform spherical morphology of T‐FeS/MoS_2_@NC is concentrated in the size range of 800–2000 nm (Figure 2c). Meanwhile, the TEM image further reveals the ordered triple shells within the T‐FeS/MoS_2_@NC sphere (Figure 2d; Figure S12, Supporting Information). The thick of nitrogen‐doped carbon layer was measured as ≈ 18 nm (Figure 2e). Thermogravimetric analysis (TGA) was applied to study the chemical change process and carbon content in the FeS/MoS_2_@NC nanocomposite under air atmosphere, as shown in Figure S13 (Supporting Information). Analysis results reveal that the increase weight (2.63 wt.%) below 345 °C is associated with the partial chemical reactions of the FeS converting to FeSO_4_. In addition, the conversion of remaining FeS and FeSO_4_ to Fe_2_O_3_, as well as MoS_2_ to MoO_3_ related to the weight reduction of 5.3 wt.%, while the significant weight loss (≈ 8.27 wt.%) occurs at 550 °C might be attributed to the carbon content in the composite.^[^ ^32^ ^]^ Figure 2f suggests that there may be slightly thickness variations among the different shell layers within the spheres. This uniformity in shell formation is crucial for maintaining the structural integrity and enhancing the electrochemical performance of the anode.^[^ ^33^, ^34^ ^]^ As shown in Figure 2g,h, the clear and equidistant lattice fringes in HRTEM images indicate that the lattice fringes of FeS and MoS_2_ are closely adhered, due to the heterostructure being obtained by the in situ sulfidation of Fe_2_(MoO_4_)_3_. Efficient heterostructures can provide more active sites and stable built‐in electric field, significantly accelerating the kinetics of Na⁺ transfer.^[^ ^35^ ^]^ As shown in Figure 2i, all diffraction rings in the SAED pattern are correspond to the crystalline planes of FeS and MoS_2_. As well, all constituent elements are detectable in the EDS mapping, confirming the uniform distribution of FeS and MoS_2_ within the material (Figure 2j). For comparison, single‐shell FeS/MoS_2_@NC spheres (S‐FeS/MoS_2_@NC) were also synthesized by adjusting the aging time of precursor. As shown in Figures S14 and S15 (Supporting Information), the single‐shell FeS/MoS_2_@NC (S‐FeS/MoS_2_@NC) exhibits the same morphology with a reduced number of shell layers compared to the T‐FeS/MoS_2_@NC. The inductively coupled plasma optical emission spectroscopy (ICP‐OES) analysis results of the FeS/MoS_2_@NC material reveal that the proportion of FeS and MoS_2_ is ≈ 5:7 (Table S1, Supporting Information).

The crystalline phase of T‐FeS/MoS_2_@NC and S‐FeS/MoS_2_@NC was analyzed by powder X‐ray diffraction (Figure S16, Supporting Information). The diffraction peaks of T‐FeS/MoS_2_@NC and S‐FeS/MoS_2_@NC all correspond well to MoS_2_ and FeS respectively, further indicating the co‐existed of crystalline FeS and MoS_2_ in the heterostructure. Then, XPS was used to study the surface composition of T‐FeS/MoS_2_@NC (Figure S17, Supporting Information). In the high‐resolution Fe 2*p* XPS peaks (Figure S17a, Supporting Information), signals at ≈ 710.5/724.1 eV and 711.9/726.3 eV are attributed to Fe^3+^ and Fe^2+^ of Fe 2*p*_1/2_/2*p*_3/2_, respectively.^[^ ^36^ ^]^ The peak area corresponding to Fe^2+^ is significantly larger than that of Fe^3+^, indicating that the predominant valence state of Fe in the material is Fe^2+^. The presence of Fe^3+^ may result from the slight oxidation of FeS, which is commonly observed in previous studies. Two main peaks in Mo 3*d* spectra (Figure S17b, Supporting Information) at ≈ 231.8 and 228.6 eV correspond to Mo 3*d*_3/2_ and Mo 3*d*_5/2_, respectively. Additionally, a weak peak at ≈ 225.8 eV is associated with the 2*s* of sulfur,^[^ ^37^ ^]^ and a peak at ≈ 235.1 eV is attributed to Mo^6+^. In the high‐resolution S 2*p* XPS spectra, two major peaks at ≈ 161.5 and 162.8 eV correspond to the 2*p*_3/2_ and 2*p*_1/2_ electronic orbitals of sulfur, respectively, indicating that sulfur exists in the S^2–^ state as shown in Figure S17c (Supporting Information). For the Raman spectrum (Figure S18, Supporting Information), both T‐FeS/MoS_2_@NC and S‐FeS/MoS_2_@NC exhibit two peaks of D band and G band located at 1345 and 1595 cm^−1^ respectively, which is related to the surface carbon shell derived from PDA pyrolysis. The calculated I_D_/I_G_ values of T‐FeS/MoS_2_@NC and S‐FeS/MoS_2_@NC are 0.91 and 0.79, indicating that graphite carbon accounts for the majority of the NC coating.^[^ ^38^ ^]^

**Figure 3:**
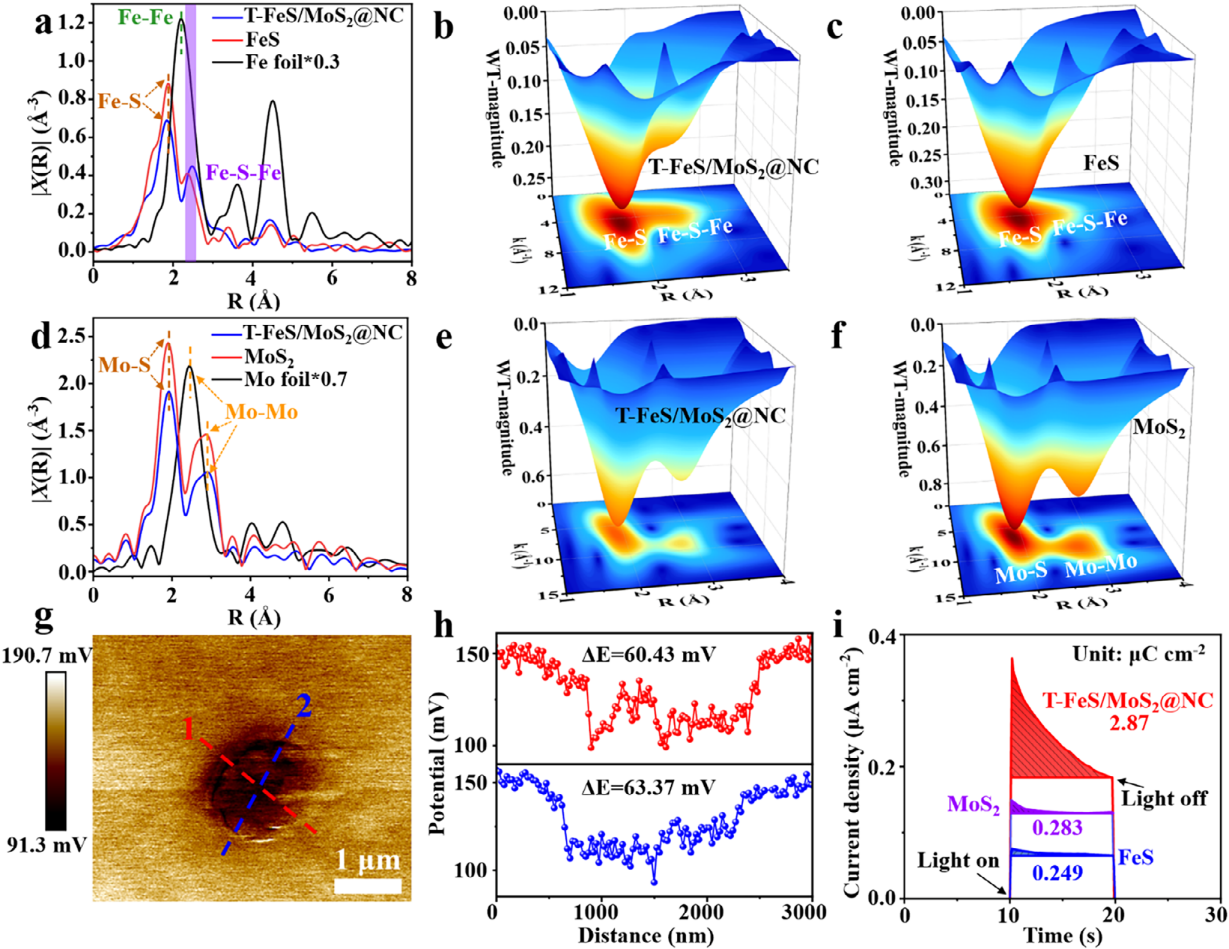
a) R‐space EXAFS analysis of Fe in Fe foil, FeS, and T‐FeS/MoS_2_@NC. b,c) WT transform profiles of Fe K‐edge for T‐FeS/MoS_2_@NC, and FeS. d) R‐space EXAFS analysis of Mo in Mo foil, MoS_2_, and T‐FeS/MoS_2_@NC. e,f) WT transform profiles of Mo K‐edge for T‐FeS/MoS_2_@NC, and MoS_2_. g) KPFM image of T‐FeS/MoS_2_@NC and h) corresponding surface potential distribution. (i) Transient photocurrent density measurements of T‐FeS/MoS_2_@NC, FeS, and MoS_2_.

To further illustrate the presence of the built‐in electric field in the T‐FeS/MoS_2_@NC composites, valence band XPS measurements were conducted (Figure S19, Supporting Information). The VB spectrum of T‐FeS/MoS_2_@NC exhibits a shape similar to that of MoS_2_. The edge energy order is as follows: T‐FeS/MoS_2_@NC (1.24 eV) < FeS (1.36 eV) < MoS_2_ (1.43 eV). The shift in the valence band position indicates the formation of a built‐in electric field in the heterostructure junction,^[^ ^39^ ^]^ resulting in band bending and enhanced conductivity as calculated in work function (Figure 1d). Synchrotron X‐ray absorption spectroscopy (XAS) was performed to probe the fine electronic structure of Fe and Mo species. The Fourier transform extended X‐ray absorption fine structure (FT‐EXAFS) spectra of the Fe K‐edge exhibit a significant shift in the Fe‐S‐Fe bond‐related peak at ≈ 2.36 Å (**Figure** **3**a), which can be attributed to the change of electron structure due to the formation of built‐in electric field.^[^ ^40^ ^]^ In the wavelet transform EXAFS (WT‐EXAFS) profile, the maximum intensity of FeS appears at k = 4.45 Å^−1^, while the maximum intensity of T‐FeS/MoS_2_@NC shows a positive shift (Figure 3b,c). This finding further supports that built‐in electric field in T‐FeS/MoS_2_@NC heterogeneous interface drives the change of Fe electron configuration.^[^ ^41^ ^]^ Similarly, the built‐in electric field in the T‐FeS/MoS_2_@NC composite also impacts the electron structure of Mo atoms. The FT‐EXAFS spectrum of the Mo K‐edge reveals a shift in the peak corresponding to the Mo–S bond compared to MoS_2_ (Figure 3d). Additionally, the WT‐EXAFS profile shows that the position of the highest intensity in T‐FeS/MoS_2_@NC is shifted relative to MoS_2_ (Figure 3e,f). The wavelet transforms of Fe foil and Mo foil are presented for comparison in Figures S20 and S21 (Supporting Information). These observations indicate that the built‐in electric field generated by the FeS/MoS_2_ heterostructure alters the electron structure of Fe and Mo atoms, thereby accelerating electrochemical reactions and further enabling excellent sodium‐ion storage kinetics.^[^ ^42^ ^]^

The surface potential of the samples was clearly visualized using Kelvin probe force microscopy (KPFM) (Figure 3g,h). The T‐FeS/MoS_2_@NC composite exhibited an average surface potential of 61.9 mV, attributed to the regulation of surface charge density by the built‐in electric field in the heterostructure.^[^ ^43^ ^]^ Consistent results were obtained from the Zeta potential analysis (Figure S22, Supporting Information), where the Zeta potential of T‐FeS/MoS_2_@NC (–18.2 mV) was significantly lower than that of FeS (–6.8 mV) and MoS_2_ (–10.5 mV). This reduction is ascribed to the redistribution of surface charge driven by the built‐in electric field.^[^ ^44^ ^]^ Additionally, a more negative surface charge enhances the attraction of Na^+^, thereby T‐FeS/MoS_2_@NC facilitates the aggregation and the rapid transportation of Na^+^. Transient photocurrent measurements were also conducted (Figure 3i), revealing that the cumulative surface charge density of T‐FeS/MoS_2_@NC reached 2.87 µC cm^−2^. This result demonstrates that the engineered built‐in electric field effectively enhances charge separation efficiency.^[^ ^45^ ^]^ This efficient charge separation further underscores the role of the built‐in electric field in enhancing the electrochemical performance of T‐FeS/MoS_2_@NC as a sodium‐ion battery anode material.

**Figure 4:**
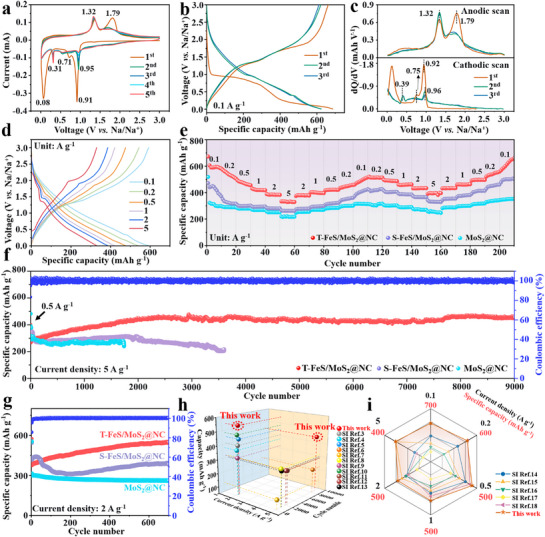
a) CV curves for the first five cycles at a scan rate of 0.1 mV s^−1^, b) charge–discharge profiles at 0.1 A g^−1^, c) dQ/dV curves for the first three cycles, and d) charge–discharge profiles at different current densities of T‐FeS/MoS_2_@NC electrode. e) Rate performance of different electrodes. f) Long‐term cycling performance of T‐FeS/MoS_2_@NC electrode at 5.0 A g^−1^. g) Cycling performance of different electrodes at 2 A g^−1^. h) Comparison of cycling stability and specific capacity, and (i) comparison of rate performance with other MoS_2_ or FeS composites.

**Figure** **4**a shows the cyclic voltammetry (CV) curves of the first five cycles for the T‐FeS/MoS_2_@NC. The cathodic peak at 0.95 V in the first cycle corresponds to the insertion of Na⁺ both into FeS and MoS_2_, forming a sodium‐rich metal sulfide phase (Na_x_FeS and Na_x_MoS_2_).^[^ ^46^, ^47^ ^]^ The peak at 0.71 V is associated with the formation of the SEI film, which is further confirmed by the subsequent DRT analysis of the impedance curve. The peak near 0.1 V is attributed to the formation of Fe^0^ and Mo^0^. In subsequent cycles, the reduction peak shifts to a higher potential (0.31 V), while the anodic peaks at 1.32 and 1.79 V correspond to the re‐oxidation of Fe and Mo, respectively. The subsequent anodic and cathodic scans show a high degree of overlap, indicating the good reversibility of the material. It is noteworthy that the anodic peak at 0.91 V decreases in intensity over the first five cycles,^[^ ^48^ ^]^ which may indicate that more sodium‐rich Na_x_FeS is formed during the oxidation of Fe^0^. In the constant current charge–discharge curves (Figure 4b), a significant irreversible capacity loss (661.3/685.9 mAh g^−1^) was observed during the first cycle, which is attributed to the formation of the SEI film and incomplete sodium removal caused by electrode structure rearrangement.^[^ ^49^ ^]^ The corresponding dQ/dV curves (Figure 4c) show that the voltage plateau closely aligns with the CV curve. As shown in Figure 4d, the charge–discharge curves at different current rates confirmed the significant electrochemical activity of T‐FeS/MoS_2_@NC, even at high current densities. Rate performance was evaluated at varying current densities of 0.1, 0.2, 0.5, 1.0, 2.0, and 5.0 A g^−1^. Compared to MoS_2_ and S‐FeS/MoS_2_@NC, T‐FeS/MoS_2_@NC exhibited superior performance, with specific capacities of 590.1, 538.1, 468.9, 419.6, 385.0, and 329.3 mAh g^−1^ at these respective current densities. Upon returning to a current density of 0.1 A g^−1^, it maintained a capacity of 515.3 mAh g^−1^ for T‐FeS/MoS_2_@NC, demonstrating an excellent rate performance (Figure 4e).

To better illustrate the advantages of heterostructures and multi‐shell nanostructures, the three different electrodes were tested at a high current density of 5 A g^−1^, as shown in Figure 4f. Benefiting from the efficient heterostructure and multi‐shell structure, T‐FeS/MoS_2_@NC maintained a high specific capacity of 451.5 mAh g^−1^ after ultralong life‐span of 9000 cycles. In contrast, MoS_2_@NC electrode showed a significant decrease in capacity after 1725 cycles, due to their inability to withstand the volume changes caused by Na⁺ insertion/extraction. The S‐FeS/MoS_2_@NC electrode experienced a considerable drop in capacity after 3500 cycles, further confirming the superiority of T‐FeS/MoS_2_@NC. Additionally, T‐FeS/MoS_2_@NC was also tested at an ultra‐high current density of 25 A g^−1^, and it also can maintain a reversible capacity of 246.1 mAh g^−1^ after 4000 cycles (Figure S23, Supporting Information). Furthermore, T‐FeS/MoS_2_@NC, MoS_2_ and S‐FeS/MoS_2_@NC were used as comparison to investigate the effects of heterostructure and shell number on electrochemical performance. At a current density of 2 A g^−1^, the T‐FeS/MoS_2_@NC exhibited a higher initial specific capacity of 408 mAh g^−1^ from the 50th cycle, with a higher specific capacity during the subsequent 700 cycles (Figure 4g), indicating an excellent cycling stability compared to the other electrodes. In conclusion, T‐FeS/MoS_2_@NC demonstrates excellent long‐cycle stability, specific capacity, and rate performance, outperforming most of the previously reported MoS_2_ or FeS composites anode materials (Figure 4h,i; Tables S2 and S3, Supporting Information).

To further investigate the excellent cycling stability of T‐FeS/MoS_2_@NC, the morphology of the anode was examined after 100 cycles at 0.5 A g^−1^. As shown in Figure S24 (Supporting Information), the material retained its intact spherical shell structure. This stability can be attributed to the abundant pores on the shell's surface, which effectively mitigate the stress induced by volume expansion during cycling. Additionally, the outer carbon coating serves to prevent structural breakage that may occur during the charge–discharge processes. The hollow structure and NC coating not only provide a large specific surface area and abundant storage sites but also create a 3D point‐to‐point network, enabling rapid charge transfer.^[^ ^50^ ^]^ In contrast, the T‐FeS/MoS_2_ anode, without a nitrogen‐doped carbon layer, maintained a reversible capacity of 385.3 mAh g^−1^ after 350 cycles at a current density of 1 A g^−1^, which is lower than that of T‐FeS/MoS_2_@NC of 419.6 mAh g^−1^ (Figure S25, Supporting Information).

**Figure 5:**
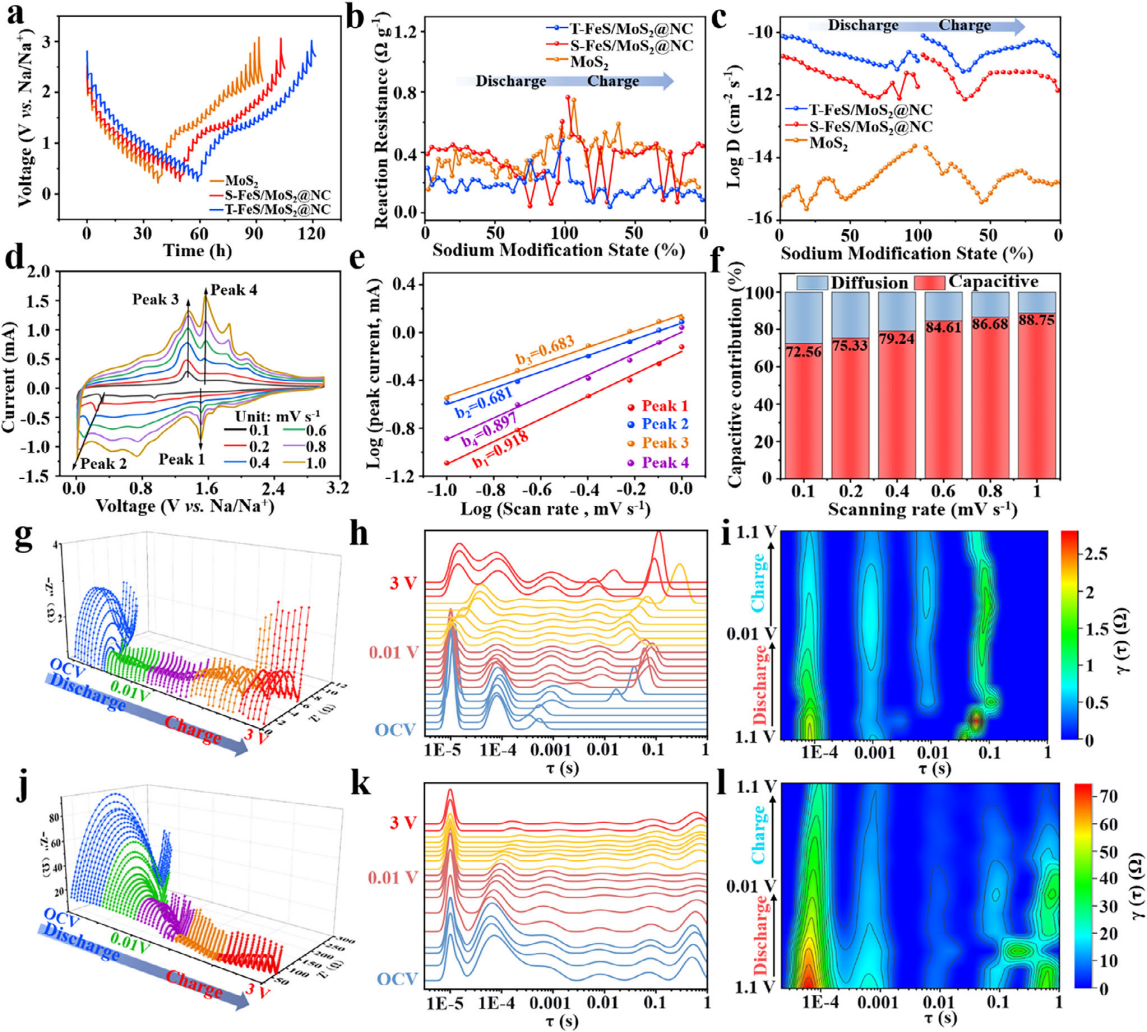
a) GITT curves of T‐FeS/MoS_2_@NC, S‐FeS/MoS_2_@NC, and MoS_2_ electrodes. (b) Calculated reaction resistance and c) *D*_Na⁺_ during charge–discharge cycles. d) CV curves of T‐FeS/MoS_2_@NC at different scan rates. e) Corresponding log (*i*) *vs* log (*v*) plots for T‐FeS/MoS_2_@NC. (f) Pseudocapacitive contribution of T‐FeS/MoS_2_@NC at different scan rates. In situ Nyquist plots of g) T‐FeS/MoS_2_@NC and j) MoS_2_ in the first cycle, h,k) DRT distribution maps resolved from in situ EIS and i,l) The fitting results of DRT in the discharge range of 1.1–0.01 V for T‐FeS/MoS_2_@NC and MoS_2_, respectively.

The diffusion coefficients (*D*_Na⁺_) and reaction resistance of T‐FeS/MoS_2_@NC, S‐FeS/MoS_2_@NC, and MoS_2_ were measured using galvanostatic intermittent titration technique (GITT) to investigate the enhancement of sodium ion transport kinetics by the heterogeneous structure.^[^ ^51^ ^]^ After three cycles of the electrodes at 0.1 A g^−1^, a 120–min pulse was applied, followed by a 20–min relaxation period at 0.05 A g^−1^ (**Figure** **5**a). As shown in Figure 5b, the T‐FeS/MoS_2_@NC electrode exhibits lower reactive resistance during charging and discharging, which is attributed to the reduction in internal resistance of the material resulting from the parallel configuration of multiple shells, which enhances electron transport and reduces internal resistance, leading to better overall performance during cycling. The *D*_Na⁺_ values can be calculated using the following formula:

(1) $$D_{Na^{+}}=\frac{4}{\pi \tau }{\left(\frac{mV_{m}}{\mathrm{MA}}\right)}^{2}{\left(\frac{\Delta E_{s}}{\Delta E_{\tau }}\right)}^{2}$$

where τ represents the duration of the pulse, and *m*, *V*_m_, and *M* denote the mass, molar volume, and molar mass of the active material, respectively.^[^ ^52^ ^]^ The *A* represents the area of the electrode. The *ΔE*_s_ refers to the change in voltage during the equilibrium state, occurring before and after the current pulse, while *ΔE*_τ_ denotes the voltage change caused by the constant current charge–discharge processes (Figure S26, Supporting Information). The respective *D*_Na⁺_ values for the T‐FeS/MoS_2_@NC, S‐FeS/MoS_2_@NC, and MoS_2_ electrodes are shown in Figure 5c. The analysis indicates that the significant change in log *D*_Na⁺_ for T‐FeS/MoS_2_@NC (10^−10^–10^−12^ cm^2^ s^−1^) and MoS_2_ (10^−14^–10^−15.5^ cm^2^ s^−1^) electrodes, implying that heterostructure engineering is beneficial for enhancing Na^+^ diffusion kinetics.^[^ ^53^ ^]^

Subsequently, CV tests of different electrodes were performed at various scan rates. Figure 5d shows the CV curve profiles of T‐FeS/MoS_2_@NC at scan rates from 0.1 to 1.0 mV s^−1^. The contribution of pseudo capacitance can be quantitatively determined by examining the relationship between current (*i*) and scan rate (*v*) using the equations *i* = *av^b^* and log (*i*) *=* log (*a*) + *b* log (*v*).^[^ ^54^ ^]^ When the value of *b* equals 0.5, it indicates that the process is primarily diffusion‐controlled, while the *b* is 1.0, it suggests that the behavior is mainly governed by pseudo capacitance.^[^ ^55^ ^]^ As shown in Figure 5e and Figure S27 (Supporting Information), the *b* values of T‐FeS/MoS_2_@NC, S‐FeS/MoS_2_@NC, and MoS_2_ are between 0.5 and 1.0, indicating that the Na⁺ storage reaction is influenced by both diffusion control and pseudo capacitance. Moreover, the results showed that as the scan rate increased, the proportion of pseudo capacitance contribution of T‐FeS/MoS_2_@NC to the total capacity gradually increased, reaching 88.75% at 1.0 mV s^−1^ (Figure 5f), this value is higher than that of 81.84% for S‐FeS/MoS_2_@NC and 80.59% for MoS_2_. This significant capacitance contribution is attributed to the unique hollow multi‐shell structure.

Furthermore, as shown in Figure S28 (Supporting Information), the Na⁺ storage performance of T‐FeS/MoS_2_@NC was tested in ether‐based electrolytes at varying current densities. The capacity and stability were both lower compared to the performance in NaPF_6_ electrolytes. This difference is likely due to the formation of SEI films with higher conductivity, such as NaF, in the NaPF_6_ electrolytes.^[^ ^56^ ^]^ As illustrated in Figure 5g,j, in situ EIS measurements were performed on T‐FeS/MoS_2_@NC and MoS_2_ electrodes to assess the impact of heterostructures on impedance values during the initial charge–discharge process (Figure S29, Supporting Information provides data for the S‐FeS/MoS_2_@NC electrode). The electrochemical processes were analyzed using the Distributed Relaxation Time (DRT) technique as shown in Figure 5h,k. The analysis revealed significant shifts in the peak locations, demonstrating distinct Na storage behaviors between the T‐FeS/MoS_2_@NC and MoS_2_ electrodes in SIBs.^[^ ^57^ ^]^ To further demonstrate the impedance changes of the product at low voltage, a contour map of T‐FeS/MoS_2_@NC was constructed and combined with the DRT results of the first charge–discharge cycle of MoS_2_ between 0.01 and 1.1 V (Figure 5i,l), with a larger time‐range linear graph shown in Figure S30 (Supporting Information). It can be observed that throughout the entire low‐voltage cycle, the impedance of the T‐FeS/MoS_2_@NC electrode (0–5.4 Ω) is much lower than that of the MoS_2_ electrode (0–137.2 Ω), and also lower than that of the S‐FeS/MoS_2_@NC electrode (0–14.8 Ω). The reduction in electrode impedance is attributed to the parallel connection of different shell layers, which further reduces the material impedance during charging and discharging. Especially, in Figure 5i, peak splitting is observed at a discharge voltage of 0.9 V, and a new peak appears at 0.7 V, which may correspond to the formation of the SEI film, consistent with the previous CV results. The overall impedance spectra exhibit no dramatic changes, while the DRT analysis reveals a pronounced increase in the signal at τ = 0.1 s specifically at 0.8 V, which is typically associated with charge‐transfer resistance (R*_ct_*) at the electrode–electrolyte interface. This transient spike in the R*_ct_*‐related time constant suggests a sudden electrochemical activation process during the initial discharge, corresponding to the formation of the SEI film.^[^ ^58^ ^]^ Additionally, as shown in Figure S31 (Supporting Information), the in situ impedance of T‐FeS/MoS_2_@NC was analyzed in ether‐based electrolytes. Compared to NaPF_6_‐based electrolytes, the ether‐based electrolyte shows more abundant impedance peaks, and the impedance values are higher (0–224.6 Ω) between 1.1 and 0.01 V, further verifying that the SEI film formed by NaPF_6_ may have stronger conductivity.

**Figure 6:**
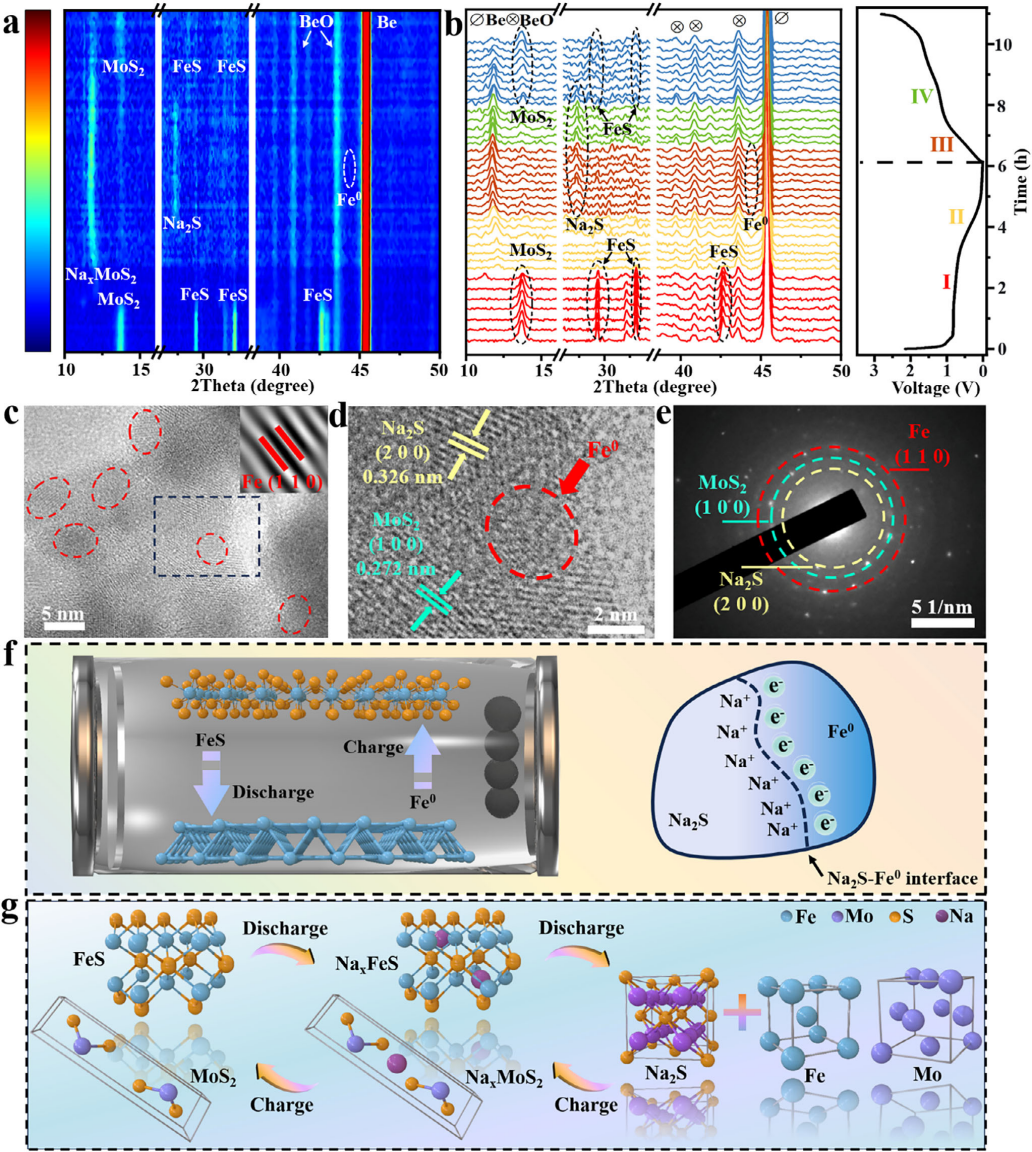
a,b) Contour plots and stacked patterns of in situ XRD patterns of T‐FeS/MoS_2_@NC in the first cycle. c,d) HRTEM images of T‐FeS/MoS_2_@NC electrode discharged to 0.2 V and e) the corresponding SAED image. f) Schematic of space charge region formation during charge–discharge process. g) Schematic diagram of the phase transition of the heterostructure during sodiation/de‐sodiation processes.

In order to gain insight into the phase transition behavior of T‐FeS/MoS_2_@NC during the first charge–discharge, T‐FeS/MoS_2_@NC was used as an electrode in a SIBs for in situ XRD measurements (**Figure** **6**a,b). It should be noted that the peaks at ≈ 38.1°, 43.5°, and 45.5° are from BeO and Be.^[^ ^59^ ^]^ At the beginning of the cell discharge, the peaks at 29.8°, 33.7°, and 43.5° are associated with the (1 0 0), (1 0 1), and (1 0 2) crystalline surfaces of FeS, while the peaks at 14.4° and 32.7° are attributed to the (0 0 2) and (1 1 0) crystal planes of MoS_2_. During the discharge process, the intensity of the diffraction peaks of FeS gradually decreased, but there was no significant change in the peak position, indicating the gradual insertion of Na^+^ into FeS to form the intermediate Na_x_FeS.^[^ ^60^ ^]^ The peak associated with Na_x_FeS gradually disappeared during subsequent discharges, while the intensity of the Na_2_S peak at 27.7° increased.^[^ ^61^ ^]^ At a discharge voltage of 0.2 V, a weak peak appeared at 44.6°, associated with Fe^0^. This is due to the fact that the generated Fe^0^ particles are extremely small in size or partially amorphous, leading to weak peak intensity. The intensity of the Na_2_S peaks gradually tapered off during the subsequent sodium removal process, and the characteristic peaks of FeS were observed again at 28.6° and 33.7°. As shown in Figure 6a, the distinct diffraction peaks associated with Na_2_S appear within the voltage window of 0.01–1.1 V. This voltage range effectively captures the electrochemical environment where Fe^0^ and Na_2_S coexist at the interface, thereby facilitating interfacial pseudocapacitive behavior. Additionally, this range encompasses the conversion reaction FeS + 2Na^+^ + 2e− → Fe^0^ + Na_2_S, which typically occurs below 0.3 V and is associated with the formation of Fe^0^ nanodots. The selected voltage window aligns well with previous studies on Fe‐based sulfides utilized for Na^+^ storage, enabling a more accurate investigation of both pseudocapacitive contributions and phase transition mechanisms.^[^ ^62^ ^]^ On the other hand, the peak at 14.4° gradually decreased during the initial stage of discharge, and the characteristic peak of Na_x_MoS_2_ appeared at 11.8°, attributed to the insertion of Na^+^ into MoS_2_. The diffraction peaks shifted back to 14.4° during the discharge process, proving the highly reversible nature of the reaction. The peaks of Mo^0^ were not observed during this process, however, in the HRTEM analysis of the discharge at 0.01 V, a lattice streak associated with Mo^0^ was found (Figure S32, Supporting Information). Interestingly, the peaks associated with Na_x_MoS_2_ were consistently present throughout the charging and discharging processes, indicating that the conversion of Na_x_MoS_2_ to Mo^0^ was not a complete reaction. This suggests that the material structure remains stable during cycling.^[^ ^63^ ^]^ In addition, the Mo atoms that are reduced during electrochemical cycling tend to aggregate into ultra‐fine nanoparticles, leading to peak broadening and signal suppression. Moreover, the metallic Mo phase likely exists in a poorly crystalline or even amorphous state, further contributing to the absence of distinct diffraction peaks.^[^ ^64^ ^]^ To further confirm the phase transition during charging and discharging, ex situ HRTEM images of the discharged (0.01 V) and charged (3.0 V) electrodes were examined. As shown in Figure S32 (Supporting Information), the lattice spacings of 0.204, 0.326, and 0.157 nm correspond to the (1 1 0), (2 0 0), and (2 0 0) crystal planes of Fe, Na_2_S, and Mo, respectively. Additionally, typical diffraction signals corresponding to the (1 1 0) and (2 1 1) crystalline phases of Fe, (2 0 0) and (4 0 0) crystalline phases of Na_2_S, and the (2 0 0) crystalline phase of Mo were observed. These results are consistent with the characterization from in situ XRD, further confirming the sodium storage mechanism of the material. Figure S33 (Supporting Information) presents the HRTEM image of the electrode after a complete charge–discharge cycle, where the lattice spacings of 0.260 and 0.271 nm correspond to the (1 1 0) crystal plane of FeS and the (1 0 1) crystal plane of MoS_2_, respectively. As shown in Figure S34 (Supporting Information), the impedance of the T‐FeS/MoS_2_@NC electrode slightly decreased after ten cycles at a current density of 1 A g^−1^. The slight decrease in impedance over the cycling period indicates the stability of the electrode material and its ability to maintain efficient charge transport during repeated charge–discharge cycles. Therefore, based on the above analysis, the sodium storage reaction mechanism of the FeS/MoS_2_ heterostructure can be expressed as described below:

(2) $$\mathrm{FeS}+\mathrm{xN}a^{+}+xe^{-}⇌Na_{x}\mathrm{FeS}$$

(3) $$\mathrm{Mo}S_{2}+\;\mathrm{xN}a^{+}+\;xe^{-}⇌\;Na_{x}\mathrm{Mo}S_{2}$$

(4) $$Na_{x}\mathrm{FeS}\;+\;(2-x)\;Na^{+}+\;(2-x)\;e^{-}⇌\mathrm{Fe}+Na_{2}S$$

(5) $$Na_{x}\mathrm{Mo}S_{2}+(4-x)\;Na^{+}+\;(4-x)e^{-}⇌\mathrm{Mo}\;+2Na_{2}S$$

The above experiments demonstrate that FeS in T‐FeS/MoS_2_@NC undergoes a phase transition to Fe^0^ when discharged to ≈ 0.3 V. To demonstrate the effect of superparamagnetic Fe nanodots on Na^+^ storage at low potentials, T‐FeS/MoS_2_@NC was used as the anode, and HRTEM imaging was performed when the half‐cell was discharged to 0.2 V (Figure 6c,d). Fe nanodots with a size of ≈ 3 nm were clearly identified, and MoS_2_ and Na_2_S were observed nearby. The diffraction rings in the SAED pattern were well attributed to Fe^0^, MoS_2_, and Na_2_S (Figure 6e). Due to the change in Fe magnetism, the spin state of Fe shifted to a high spin state, which induced high‐spin electrons to be injected into the Fe^0^ electron orbitals, exhibiting pseudocapacitive behavior at the interface with Na_2_S.^[^ ^12^ ^]^ The region around Fe^0^ nanodots had a higher concentration of Na^+^ ions and acted as an ion reservoir, efficiently transferring ions to the nearby MoS_2_ through the heterostructure, thereby accelerating the Na^+^ transport dynamics at low potentials.^[^ ^23^ ^]^

In order to clarify the sodium storage process related to Fe^0^ and explore the dynamic behavior of T‐FeS/MoS_2_@NC and MoS_2_ between 0.01 and 1.1 V, CV curves were tested at scan rates ranging from 0.1 to 1 mV s^−1^ (Figure S35, Supporting Information). It can be seen that the *b* values for FeS/MoS_2_@NC are higher than those for MoS_2_, indicating that the electrochemical behavior of the T‐FeS/MoS_2_@NC composite material at voltages between 0.01 and 1.1 V is more closely related to a capacitance‐controlled process.^[^ ^65^ ^]^ The capacitance contribution of T‐FeS/MoS_2_@NC at 1.0 mV s^−1^ is 77.44%, which is much higher than the capacitance contribution of MoS_2_ at the same scan rate (34.62%). To further validate this observation, 10 000 charge–discharge cycles were conducted on T‐FeS/MoS_2_@NC and MoS_2_ electrodes within a voltage window of 0.01–1.1 V at a current density of 10 A g^−1^. The capacity decay of T‐FeS/MoS_2_@NC during the first 4 000 cycles is attributed to changes in the voltage window, leading to incomplete conversion of Fe^0^ to FeS. However, after 10 000 cycles, T‐FeS/MoS_2_@NC retained a capacity of 54.9 mAh g^−1^, much higher than the 17.8 mAh g^−1^ capacity of MoS_2_ under the same conditions, demonstrating the effectiveness of superparamagnetic Fe nanodots in promoting stable and rapid Na^+^ transport (Figure S36, Supporting Information). Based on the aforementioned experimental observations, Fe^0^ does not exhibit significant oxidation below 1.0 V, which effectively minimizes the contribution of conventional faradaic redox reactions. This condition allows for a focused investigation of interfacial pseudocapacitive behavior. Specifically, the Fe^0^/Na_2_S interface demonstrates rapid pseudocapacitive effects that arise from spin‐polarization‐induced surface capacitance. Consequently, within the selected voltage window, charge storage is predominantly governed by interfacial pseudocapacitance rather than conventional diffusion‐limited mechanisms. As shown in Figure 6f, superparamagnetic Fe nanodots are formed during the conversion reaction of FeS, thereby generating a surface capacitance effect at the Fe/Na_2_S interface, which stores additional sodium ions and further enhances ion transport, especially at high current densities.^[^ ^66^ ^]^ The accumulation of spin‐polarized electrons on the surface and within Fe induces the migration of sodium ions toward Fe, resulting in their accumulation on the surface of Fe under the influence of an electric field. This phenomenon leads to the generation of spin‐polarized surface capacitance and the formation of a space charge region.^[^ ^12^ ^]^ This indicates that the built‐in electric field and Fe^0^ nanodots jointly promote the rapid transport of sodium ions during the charge–discharge process. Figure 6g illustrates a schematic of the phase transition in the electrode. The analysis results are in good agreement with the process observed in the in situ XRD measurements, providing preliminary confirmation of the sodium storage and conversion reaction mechanism in T‐FeS/MoS_2_@NC.

**Figure 7:**
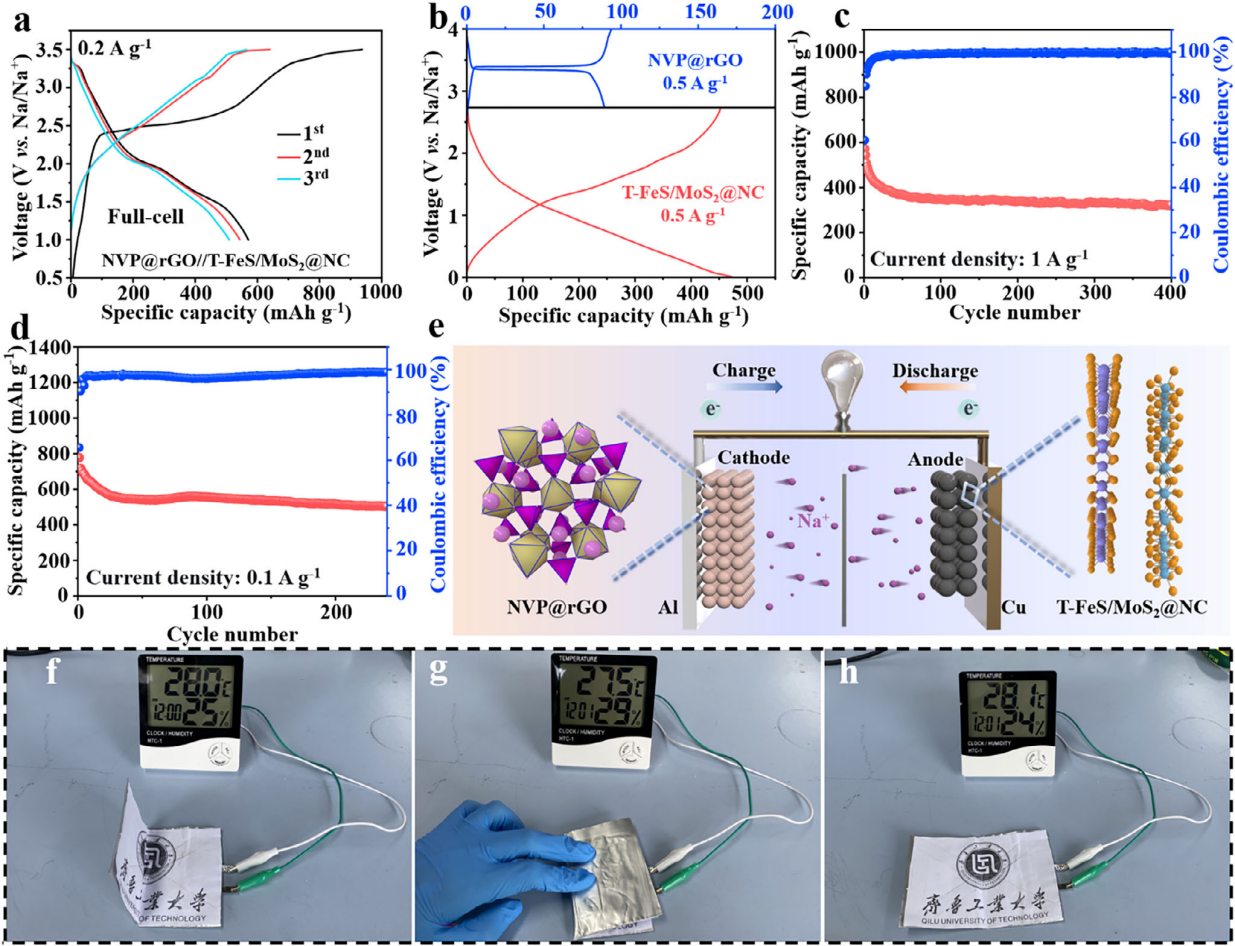
a) Charge–discharge curves of the NVP@rGO//T‐FeS/MoS_2_@NC coin‐type full‐cell at 0.2 A g^−1^. b) Half‐cell charge–discharge curves of NVP@rGO and T‐FeS/MoS_2_@NC at 0.5 A g^−1^. Coin‐type full‐cell cycling performance at c) 1 A g^−1^ and d) 0.1 A g^−1^, e) Schematic illustration of the NVP@rGO//T‐FeS/MoS_2_@NC full‐cell structure. f) A pouch full‐cell of NVP@rGO//T‐FeS/MoS_2_@NC power a household thermometer.

To evaluate the potential application of T‐FeS/MoS_2_@NC electrodes, coin‐type sodium‐ion full batteries consisting of T‐FeS/MoS_2_@NC anode and Na_3_V_2_(PO_4_)_3_@RGO (NVP@rGO) cathode were successfully assembled and tested under a voltage window of 1.0 to 3.5 V. The XRD pattern for NVP@rGO is shown in Figure S37 (Supporting Information). **Figure** **7**a shows the charge–discharge curve of the NVP@rGO//T‐FeS/MoS_2_@NC coin‐typefull‐cell at a current density of 0.2 A g^−1^. The curves demonstrate good convergence in the first three cycles, indicating relatively good reversibility of the full cell. Figure 7b shows the charge–discharge profiles of the NVP@rGO and T‐FeS/MoS_2_@NC electrodes in their respective Na^+^ half‐cells at a current density of 0.5 A g^−1^, which provided reversible specific capacities of 453.3/472.5 and 93.9/89.1 mAh g^−1^, respectively. The coin‐type full cell achieved a capacity of  571.1 mAh g^−1^ in the first cycle at a higher current density of 1 A g^−1^, with a capacity retention of 56.4% after 400 cycles (Figure 7c). Similarly, a capacity of 778 mAh g^−1^ in the first cycle at 0.1 A g^−1^, with a capacity retention of 64.6% after 240 cycles (Figure 7d). Therefore, the schematic illustration of the full battery according to the investigation is provided in Figure 7e. Furthermore, a pouch full cell was successfully fabricated, and the household thermometer could still be used safely and effectively when the pouch full cell was bent to different angles (Figure 7f). The above electrochemical performances indicate that the full cell using T‐FeS/MoS_2_@NC anode holds great promise for practical applications.

## 3 Conclusion

In summary, we synthesized triple‐shell T‐FeS/MoS_2_@NC using an improved sequential template method, combined with structural design and built‐in electric field in heterostructure, effectively addressing the challenges mentioned earlier. As an anode material for SIBs, it demonstrates excellent cycling stability that it retains 451.5 mAh g^−1^ after 9000 cycles at 5 A g^−1^. The coin‐type full battery Na_3_V_2_(PO_4_)_3_@rGO//T‐FeS/MoS_2_@NC showcased an impressive electrochemical performance, achieving 322.2 mAh g^−1^ after 400 cycles at 1 A g^−1^. The outstanding performance can be attributed to the synergy of built‐in electric field in efficient heterostructure and HoMS, as well as the appearence of superparamagnetic Fe nanodots at low potential, which facilitate the Na storage and simultaneously improve rate performance and cycling stability. Moreover, the unique triple‐shell structure shortens the charge diffusion path, enabling rapid electrochemical reactions and high cycling stability. In situ XRD, in situ EIS, and ex situ HRTEM analysis confirmed that the phase transition during cycling is response for the Na storage mechanism. This strategy provides a new approach for the synthesis of other transition metal‐based SIBs anodes.

## Conflict of Interest

The authors declare no conflict of interest.

## Supporting information



Supporting Information

## Data Availability

The data that support the findings of this study are available from the corresponding author upon reasonable request.
