# Supplementary material for: Triple‐Shell Hollow FeS/MoS2 Heterostructure Anodes: Synergistic Effects of Built‐In Electric Field on Ultra‐Stable Sodium Storage
Source: Adv Sci (Weinh). 2025 Aug 26;12(43):e09997. doi: 10.1002/advs.202509997 (PMC12631837; doi:10.1002/advs.202509997)
Supplement: Supplementary file 1 — Supporting Information [file ADVS-12-e09997-s001.docx]

Supporting Information

Triple-Shell Hollow FeS/MoS_2_ Heterostructure Anodes: Synergistic Effects of Built-In Electric Field on Ultra-Stable Sodium Storage

Mingyang Chen,^a^ Shaonan Gu,^a,^* Junhui Li,^a^ Yuxin Dai,^a^ Yanyan He,^a,^* Bin Sun,^a^ Tingting Gao,^a^ Liqiang Xu,^b,^* Guowei Zhou ^a,^*

M. Chen, Prof. S. Gu, J. Li, Y. Dai, Dr. Y. He, Prof. B. Sun, Prof. T. Gao, Prof. G. Zhou

Key Laboratory of Fine Chemicals in Universities of Shandong,

Jinan Engineering Laboratory for Multi-scale Functional Materials,

School of Chemistry and Chemical Engineering,

Qilu University of Technology (Shandong Academy of Sciences),

Jinan 250353, P. R. China

E-mails: sngu@qlu.edu.cn, heyanyan@qlu.edu.cn, gwzhou@qlu.edu.cn

Prof. L. Xu

Key Laboratory of Colloid and Interface Chemistry,

School of Chemistry and Chemical Engineering,

State Key Laboratory of Crystal Materials,

Shandong University,

Jinan 250100, P. R. China

E-mail: xulq@sdu.edu.cn

**1. Experimental Section**

**1.1 Synthesis of Fe_2_(MoO_4_)_3_ HoMS**

Fe_2_(MoO_4_)_3_ HoMS was synthesized by a sequential template method. Initially, 39 g of sucrose was dissolved in 50 mL deionized water. The resulting solution was transferred into a Teflon-lined autoclave for hydrothermal treatment. The obtained product was subsequently washed three times with deionized water to yield carbon spheres. Then, 1.59 g (NH_4_)_2_MoO_4_, 1.56 g citric acid, and 8.1 g FeCl_3_⋅6H_2_O were added into a mixed solvent of deionized water and ethanol (v: v = 3:1). Then, carbon spheres were introduced to above solution as template, and leaving at 50 °C for 48 h. Subsequently, the black powder was separated by centrifuge and washed with water and ethanol several times. Afterwards, the collected precursor was calcined in a muffle furnace at 350 ℃ for 180 min with the heating rate of 2 ℃ min^–1^. The singel-shell Fe_2_(MoO_4_)_3_ was achieved by adjusting incubation time as 12 h under the other situation remain unchanged.

**1.2** **Synthesis of FeS/MoS_2_**

FeS/MoS_2_ was realized by sulfidizing Fe_2_(MoO_4_)_3_ in tube furnace at 750 ℃ under Ar/H_2_ (V/V 95:5), and the heating rate is 15 ℃ min^–1^. Thiourea and Fe_2_ (MoO_4_)_3_ were settled on upstream and downstream of the airflow separately in the same ceramic boat. Then, the final product FeS/MoS_2_ was obtained.

**1.3** **Synthesis of FeS/MoS_2_@NC**

Add 1.21 g of Tris to 1.0 L of deionized water, and adjust the pH by adding HCl (98 %) dropwise to obtain Tris buffer solution (pH ≈ 8.5). Finally, 200 mg FeS/MoS_2_ and 100 mg dopamine were added to 300 mL Tris solution and stirred for 24 hours to obtain FeS/MoS_2_@PDA. FeS/MoS_2_@PDA was placed in a tube furnace and calcined under Ar atmosphere at 450 ℃ for 2 hours at a heating rate of 3 ℃ min^–1^ to obtain FeS/MoS_2_@NC.

**1.4** **Synthesis of Na_3_V_2_(PO_4_)_3_@rGO**

0.0795 g Na_2_CO_3_, 0.348 g V(C_5_H_7_O_2_)_3_ and 0.173 g NH_4_H_2_PO_4_ were added to 40 mL of deionized water and then stirred to obtain a homogeneous solution. The solution was transferred to a 100 mL Teflon-lined stainless steel autoclave and heated at 180 °C for 12 h. Then, 10 mL of GO solution was added with stirring and then freeze-drying. The Na_3_V_2_(PO_4_)_3_@rGO powder was obtained by annealing above processor at 750 °C for 8 h in an Ar/H_2_ (95:5 V/V) atmosphere.

**1.5 Electrochemical measurements**

The Na storage performances of T-FeS/MoS_2_@NC, S-FeS/MoS_2_@NC and pure MoS_2_ were tested by assembling CR2032 coin cell. The active material, Super P and sodium carboxymethyl cellulose (CMC) were mixed with water in a weight ratio of 7:2:1 and ball milled to obtain a slurry. The slurry was then applied to the copper foil and dried. The copper foil was cut into circular sheets with a diameter of 12 mm, where the mass loading of active material was 0.8~1.5 mg cm^–2^. Metal Na was used as the counter and reference electrode, glass fiber membrane (Whatman GF/F) was used as the separator, and 1 M NaPF_6_ dissolved in diethylene glycol dimethyl ether (DEGDME) was used as the electrolyte. Constant-current charge-discharge tests (GCD) and constant-current intermittent titration tests (GITT) were performed on LAND battery test system (CT2001A, China). Cathode electrode was prepared as follow: Na_3_V_2_(PO_4_)_3_@rGO, Super P and polyvinylidene fluoride (PVDF) were mixed with N-methyl-2-pyrrolidone (NMP) in a mass ratio of 8:1:1 to form a slurry, which was then coated onto Al foil. The sodium ion full-cells were assembled with FeS/MoS_2_@NC anode and Na_3_V_2_(PO_4_)_3_@rGO cathode, and the capacity ratio of the FeS/MoS_2_@NC and Na_3_V_2_(PO_4_)_3_@rGO is about 1: (1.2−1.5).

**1.6 Computational details**

The DFT calculations of all the models in this work were conducted in the framework of CASTEP^1^ in BIOVIA Materials Studio 2018. The exchange-correlation functional at the generalized gradient approximation (GGA) level was described using the Perdew-Burke-Ernzerhof (PBE) functional.^2^ A semi-empirical van der Waals correction accounted for the dispersion was included through the use of the DFT-D (Grimme) method. For geometry optimization, the convergence criterion for the maximum final force was 0.03 eV Å^–1^, the maximum final displacement was 0.001 Å, and 1.0 ×10^–5^ eV/atom for the total energy of the system was utilized for all computations. The plane wave cutoff energy is 598.7 eV for adsorption calculation, and the vacuum slab was set as 15 Å in the vertical direction to avoid the interaction between the layers. The bindng energies between FeS, MoS_2_, FeS/MoS_2_ and Na was defined by the following formula: E_ad_ = (E_Total_ − E_Na_ + E_Surf_) where E_Total_ and E_Surf_ are the total energies of FeS/MoS_2_ (FeS or MoS_2_) adsorbed with and without Na, and E_Na_ is the total energy of the singel Na atom.

**Figure Captions**

**Figure S1**. The optimized configurations of Na^+^ absorbed on the surface of (a) FeS (0 0 1), (b) MoS_2_ (0 0 1), and (c) FeS/MoS_2_ heterostructure.

**Figure S2.** The calculated work function of FeS and MoS_2_.

**Figure S3**. The optimized configurations of the Na^+^ diffusion paths on the surfaces of (a) FeS, (b) MoS_2_, and (c) FeS/MoS_2_.

**Figure S4**. The Fourier-transform infrared (FTIR) spectrum of carbonaceous microsphere template.

**Figure S5**. (a) SEM image of carbon spheres and (b-d) elements distribution.

**Figure S6**. SEM images of carbon spheres calcined at a) 300 ℃ and b) 450 ℃.

**Figure S7.** XRD pattern of Fe_2_(MoO_4_)_3_ precursor.

**Figure S8**. SEM images of Fe_2_(MoO_4_)_3_ precursor at different magnifications.

**Figure S9**. Thermogravimetric analysis (TGA) curve of Fe_2_(MoO_4_)_3_ precursor during carbon spheres template removal.

**Figure S10**. SEM image of FeS/MoS_2_ hollow sphere.

**Figure S11.** TEM image of FeS/MoS_2_ hollow sphere.

**Figure S12.** TEM image of T-FeS/MoS_2_@NC.

**Figure S13.** TGA curves of FeS/MoS_2_@NC nanocomposite in air with a heating rate of 10 °C min^–1^ from room temperature to 800 °C.

**Figure S14.** SEM images of S-FeS/MoS_2_@NC at different magnifications.

**Figure S15.** TEM images of S-FeS/MoS_2_@NC at different magnifications.

**Figure S16**. XRD pattern of S-FeS/MoS_2_@NC and T-FeS/MoS_2_@NC.

**Figure S17**. High-resolution XPS spectra of T-FeS/MoS_2_@NC: (a) Fe 2*p*, (b) Mo 3*d* and (c) S 2*p*.

**Figure S18**. Raman spectra of T-FeS/MoS_2_@NC, S-FeS/MoS_2_@NC, and MoS_2_.

**Figure S19.** VB-XPS spectra of T-FeS/MoS_2_@NC, MoS_2_ and FeS.

**Figure S20.** Wavelet transform diagram of Fe foil.

**Figure S21.** Wavelet transform diagram of Mo foil.

**Figure S22**. Zeta potentials of T-FeS/MoS_2_@NC, MoS_2_ and FeS.

**Figure S23.** The cycling performance of the T-FeS/MoS_2_@NC electrode at a current density of 25 A g^–1^.

**Figure S24.** The SEM image of T-FeS/MoS_2_@NC after 100 cycles at 0.5 A g^–1^.

**Figure S25.** Cycling performance of FeS/MoS_2_ electrode material at 1 A g^–1^.

**Figure S26.** The detailed GITT diagram with a single current pulse for 1200 s followed by an open-circuit for 7200 s.

**Figure S27**. (a) CV curves, (b) corresponding log(*i*) *vs* log(*v*) plots, and (c) pseudocapacitive contributions of S-FeS/MoS_2_@NC at different scan rates. (d) CV curves, (e) corresponding log(*i*) *vs* log(*v*) plots, and (f) pseudocapacitive contributions of MoS_2_ at different scan rates.

**Figure S28.** The cycling performances of the T-FeS/MoS_2_@NC electrode material were evaluated using a carbonate-based electrolyte consisting of 1 M NaClO_4_ in a mixture of ethylene carbonate (EC), diethyl carbonate (DEC), and ethyl methyl carbonate (EMC) (1:1:1, v/v/v). The tests were conducted at current densities of (a) 1 A g^–1^, (b) 5 A g^–1^, and (c) 10 A g^–1^.

**Figure S29**. The *in-situ* impedances of the S-FeS/MoS_2_@NC electrode in the first cycle and the DRT fitting results from 1.1 V to 0.01 V.

**Figure S30.** DRT fitting results of (a) T-FeS/MoS_2_@NC and (b) MoS_2_ electrodes in the first cycle with a voltage range from 1.1V to 0.01 V.

**Figure S31.** The in-situ impedance of the T-FeS/MoS_2_@NC electrode was measured using a carbonate-based electrolyte consisting of 1M NaClO_4_ in a 1:1:1 (v/v/v) mixture of ethylene carbonate (EC), diethyl carbonate (DEC), and ethyl methyl carbonate (EMC). The DRT fitting results were obtained over a voltage range of 1.1 V to 0.01 V.

**Figure S32**. (a-c) The *ex-situ* HRTEM images and (d) the corresponding SAED pattern when T-FeS/MoS_2_@NC is discharged to 0.01V.

**Figure S33.** *Ex-situ* HRTEM image of T-FeS/MoS_2_@NC anode after being fully charged to 3.0 V.

**Figure S34**. EIS images of T-FeS/MoS_2_@NC before and after 10 cycles at a current density of 1 A g^–1^.

**Figure S35**. CV curves of (a) T-FeS/MoS_2_@NC, and (d) MoS_2_ at different scan rates within a voltage window of 0.01~1.1 V. The corresponding log (*i*) vs log (*v*) plots of (b) T-FeS/MoS_2_@NC and (e) MoS_2_. Pseudo-capacitive contributions of (c) T-FeS/MoS_2_@NC and (f) MoS_2_ at different scan rates within a voltage window of 0.01~1.1 V.

**Figure S36.** Cycling performances of T-FeS/MoS_2_@NC and MoS_2_@NC electrodes at a current density of 10 A g^–1^ within a voltage window of 0.01~1.1 V.

**Figure S37**. A typical XRD pattern of NVP@rGO.

**Table S1.** The inductively coupled plasma optical emission spectroscopy (ICP-OES) analysis results of FeS/MoS_2_@NC.

**Table S2.** Comparison of cycling stability and specific capacity between T-FeS/MoS_2_@NC and previously reported metal sulfide anodes for SIBs.

**Table S3.** Comparison of rate capacity between T-FeS/MoS_2_@NC and previously reported metal sulfide anodes for SIBs.


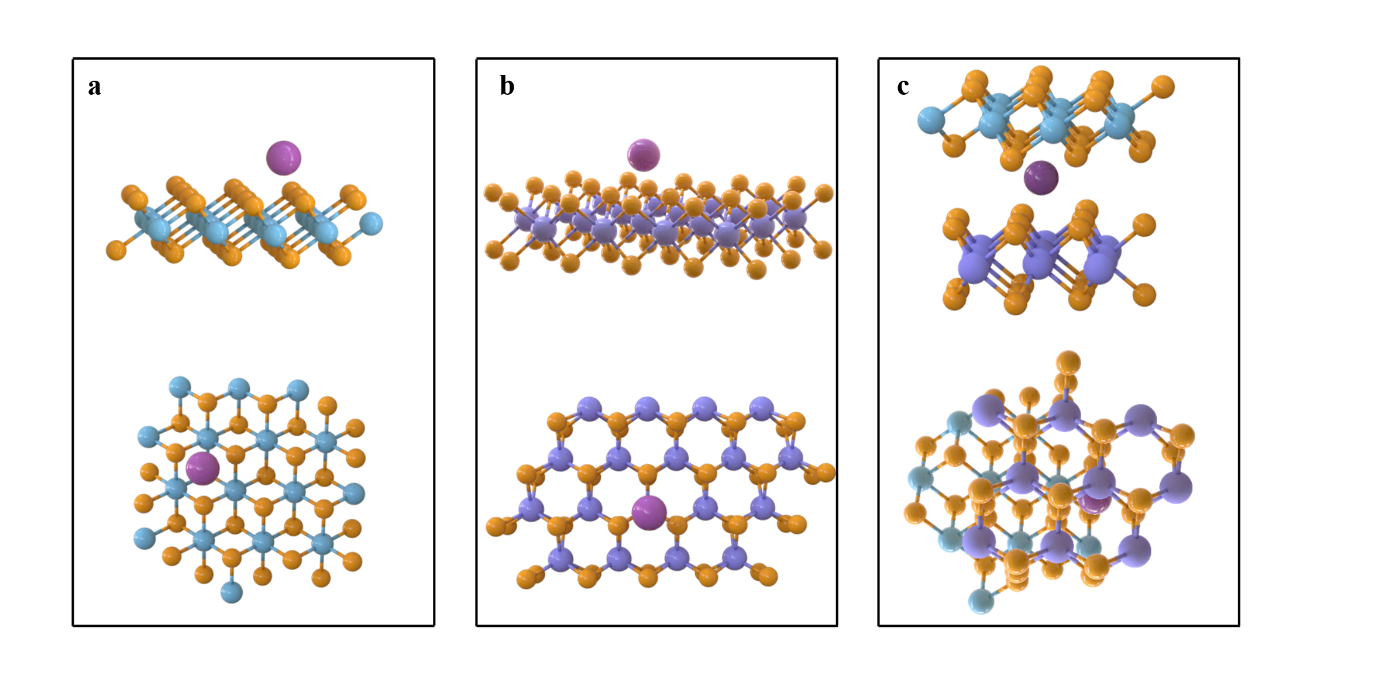


**Figure S1**. The optimized configurations of Na^+^ absorbed on the surface of (a) FeS (0 0 1), (b) MoS_2_ (0 0 1), and (c) FeS/MoS_2_ heterostructure.


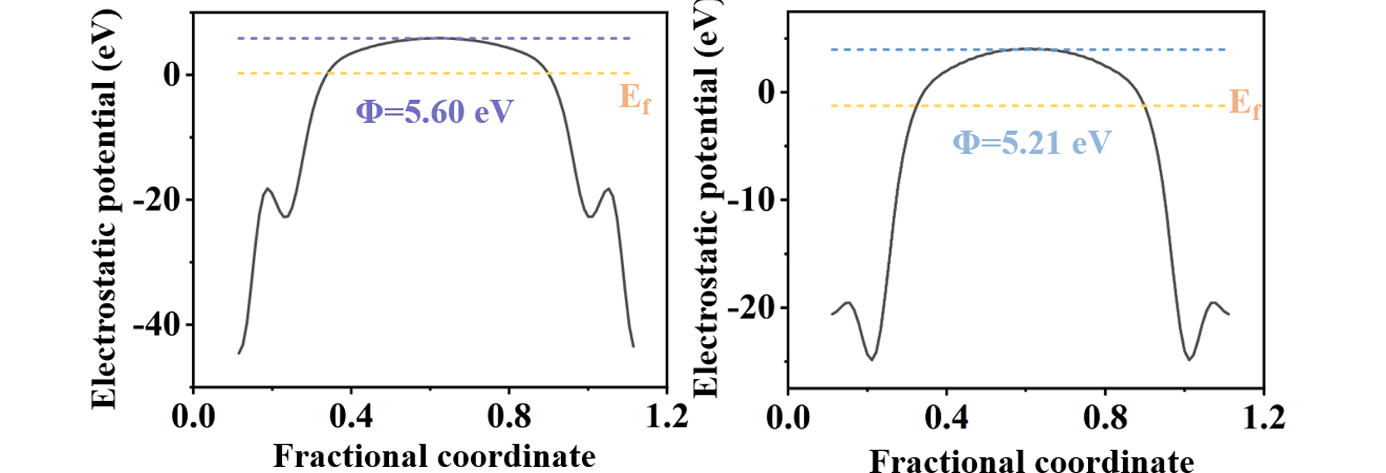


**Figure S2.** The calculated work function of FeS and MoS_2_.


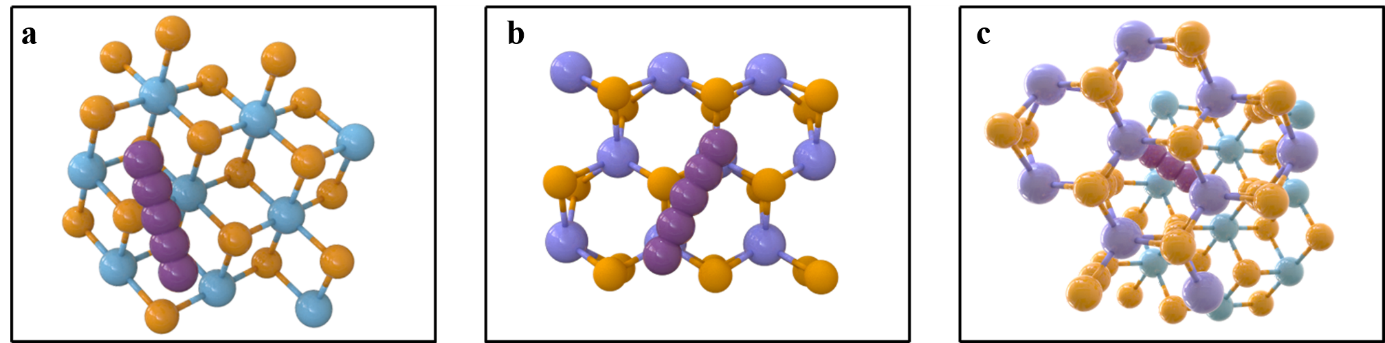


**Figure S3**. The optimized configurations of the Na^+^ diffusion paths on the surfaces of (a) FeS, (b) MoS_2_, and (c) FeS/MoS_2_.


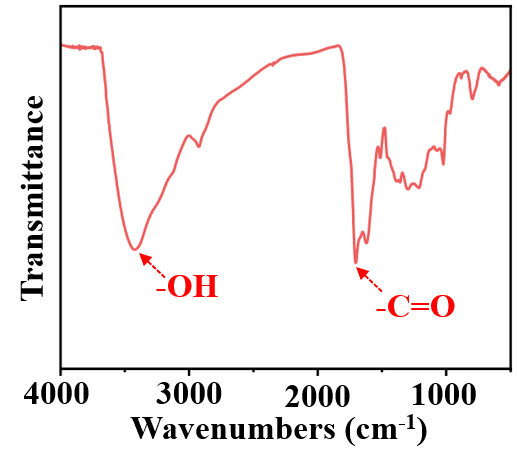


**Figure S4**. The Fourier-transform infrared (FTIR) spectrum of carbonaceous microsphere template.


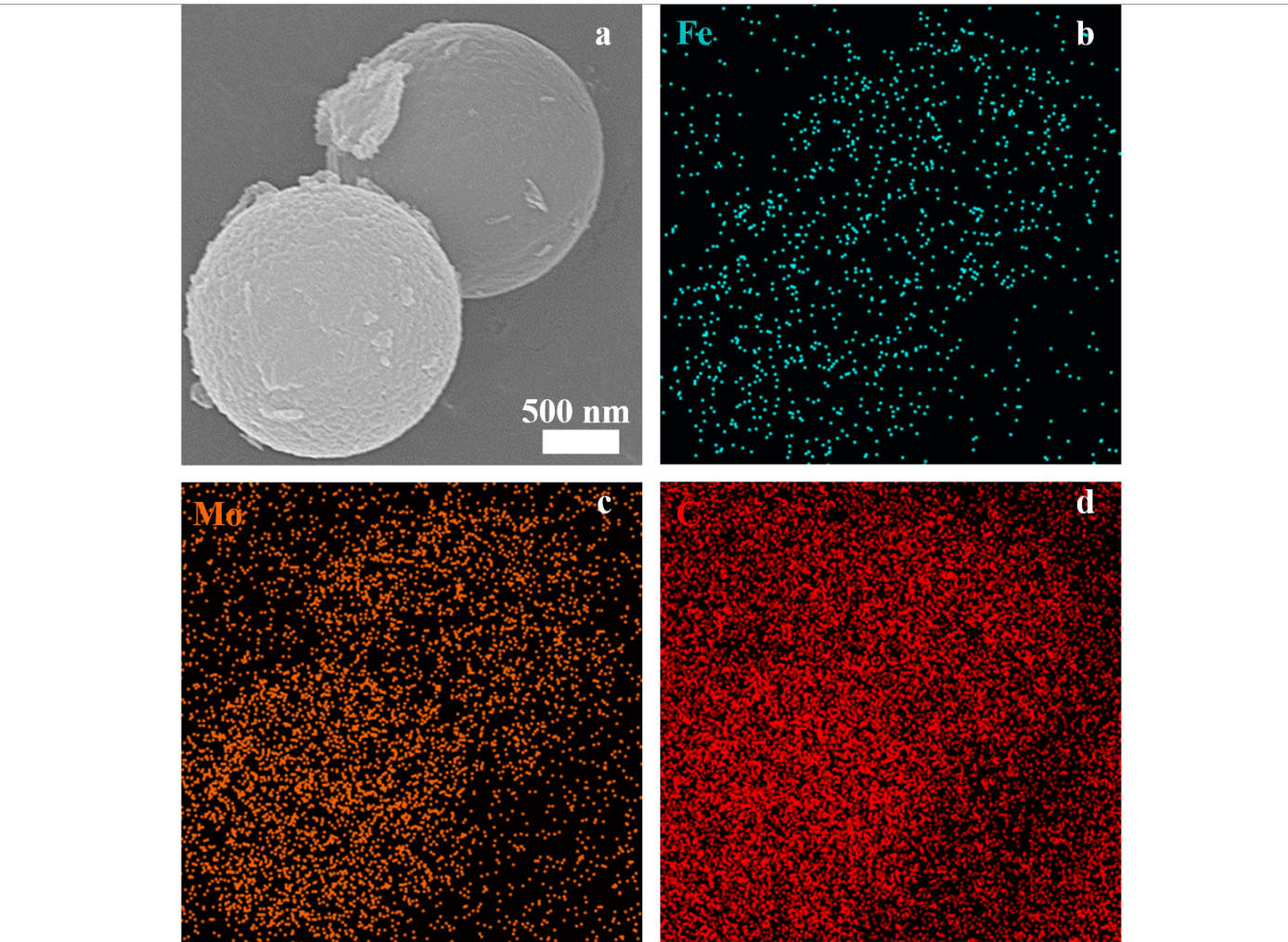


**Figure S5**. (a) SEM image of carbon spheres and (b-d) elements distribution.


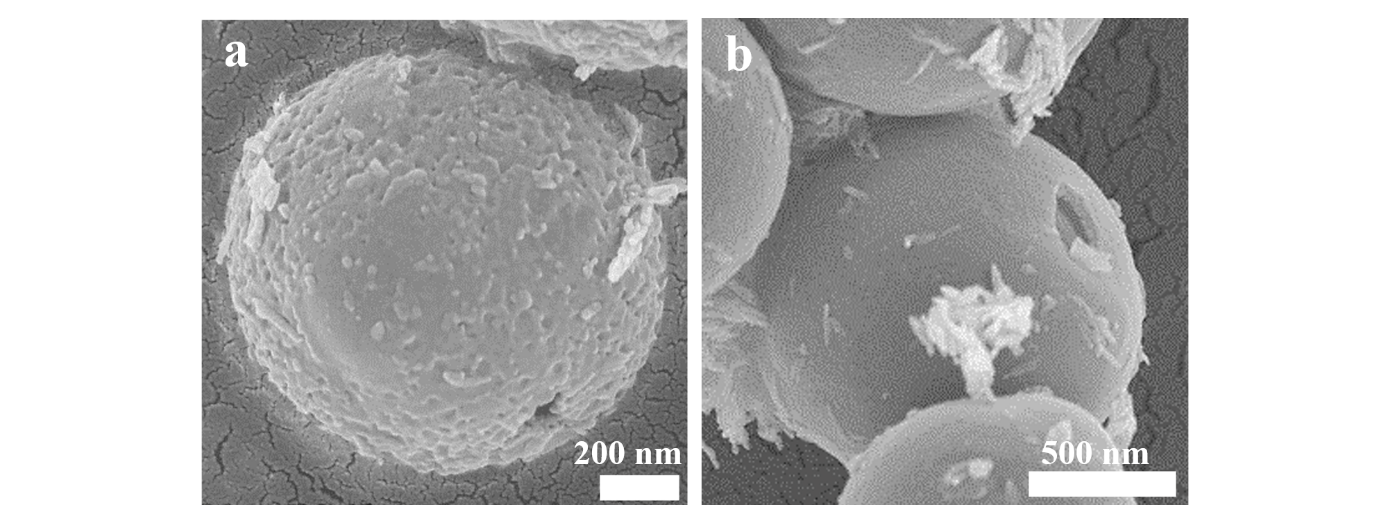


**Figure S6**. SEM images of carbon spheres calcined at a) 300 ℃ and b) 450 ℃.


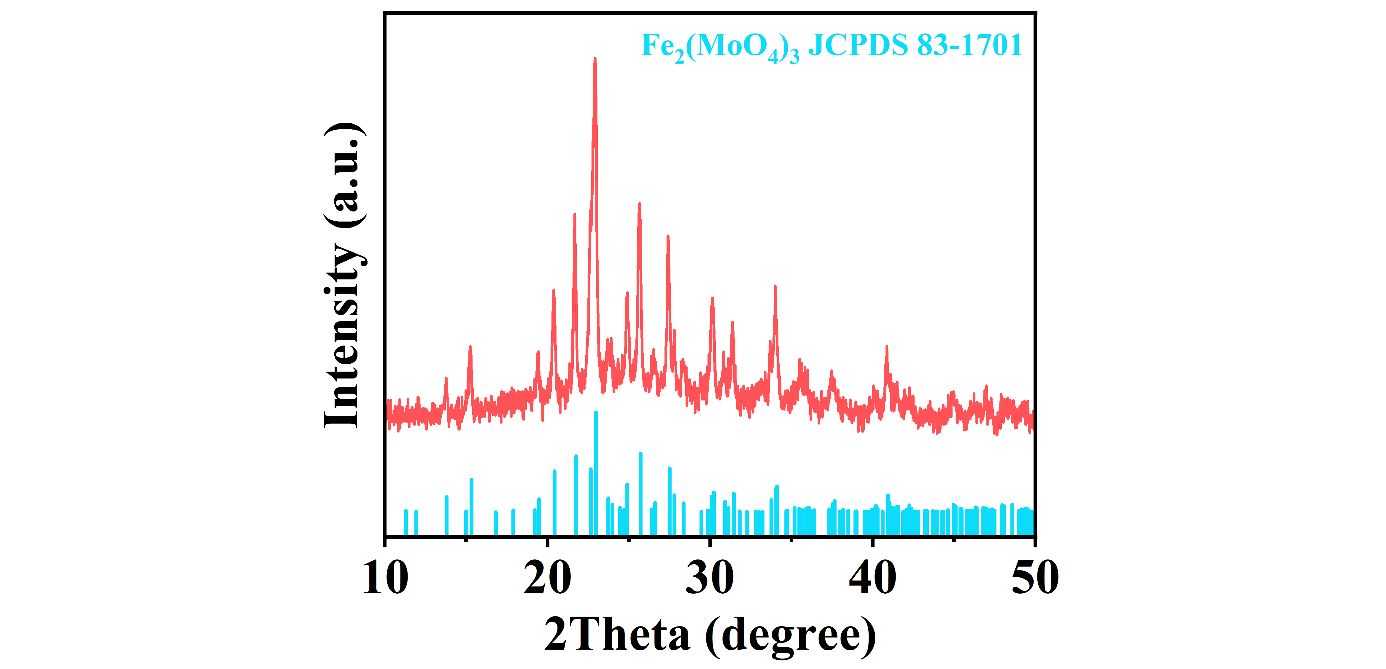


**Figure S7.** XRD pattern of Fe_2_(MoO_4_)_3_ precursor.


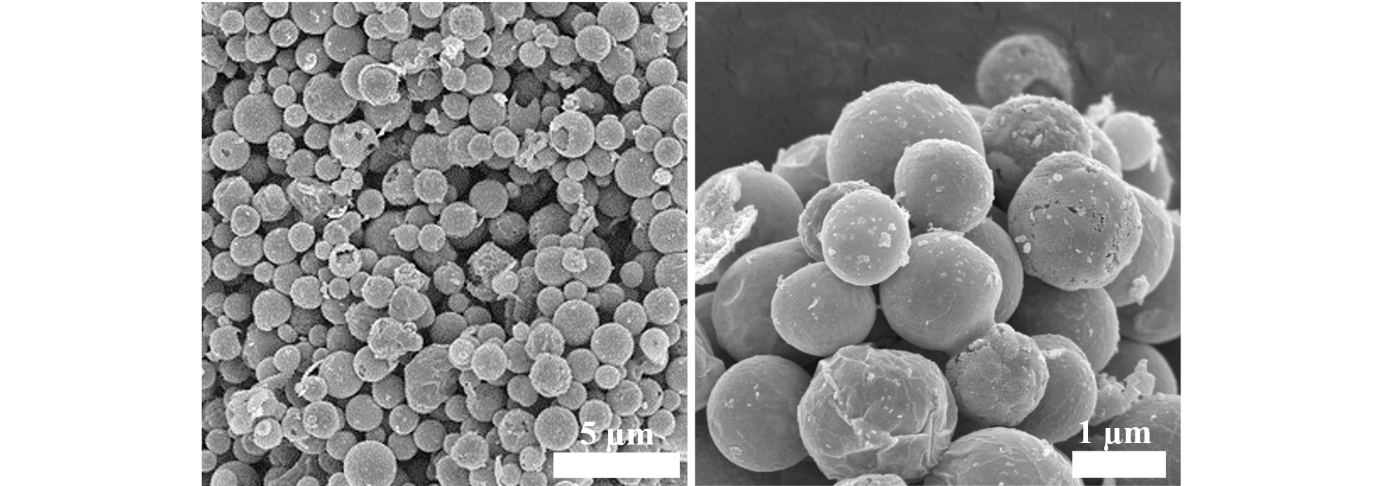


**Figure S8**. SEM images of Fe_2_(MoO_4_)_3_ precursor at different magnifications.


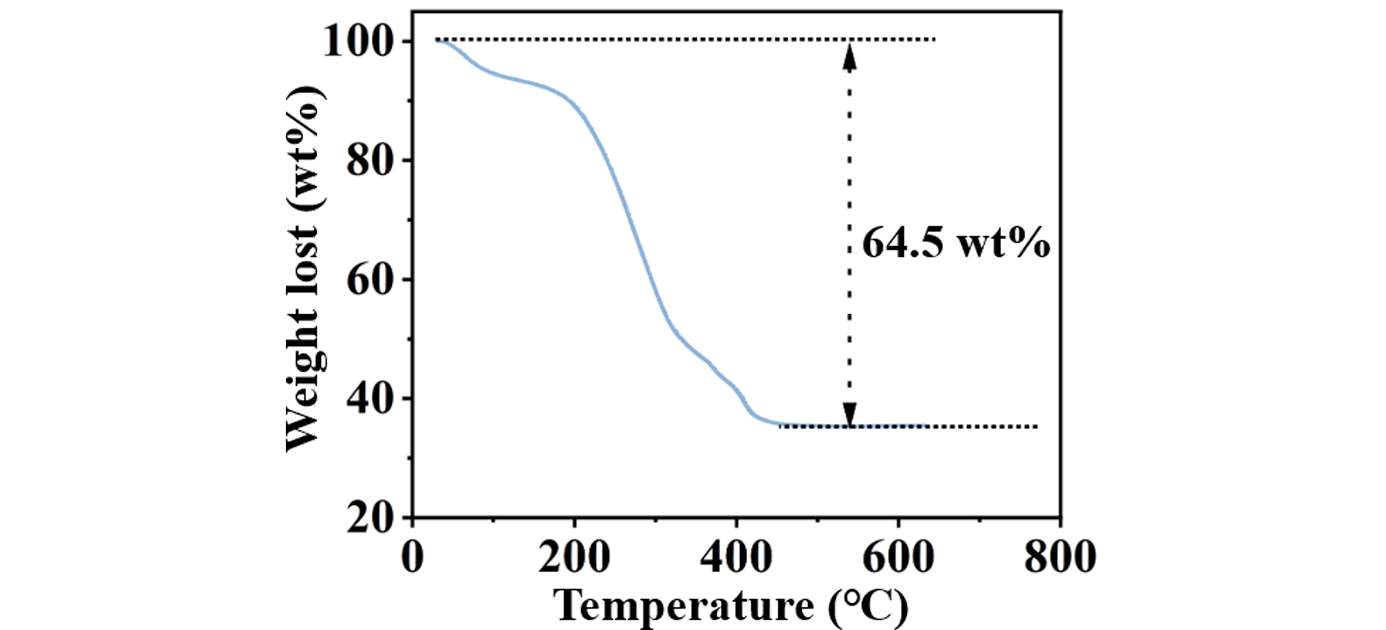


**Figure S9**. Thermogravimetric analysis (TGA) curve of Fe_2_(MoO_4_)_3_ precursor during carbon spheres template removal.


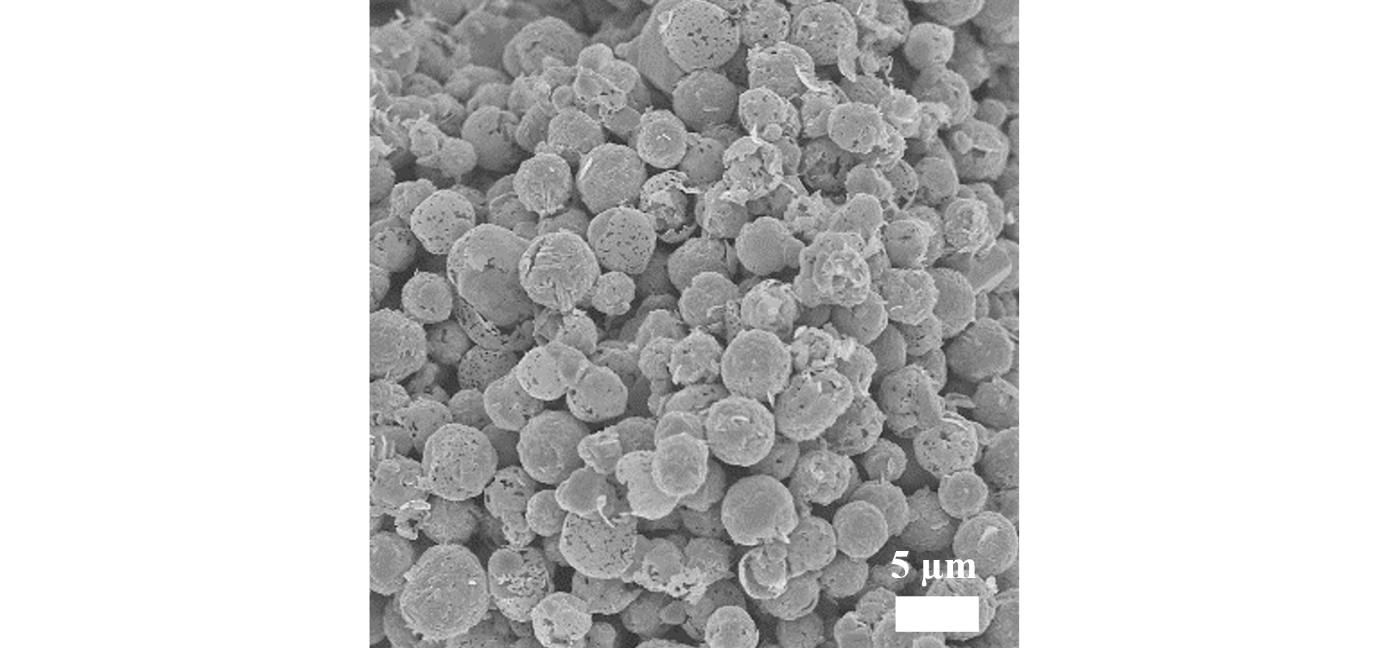


**Figure S10**. SEM image of FeS/MoS_2_ hollow sphere.


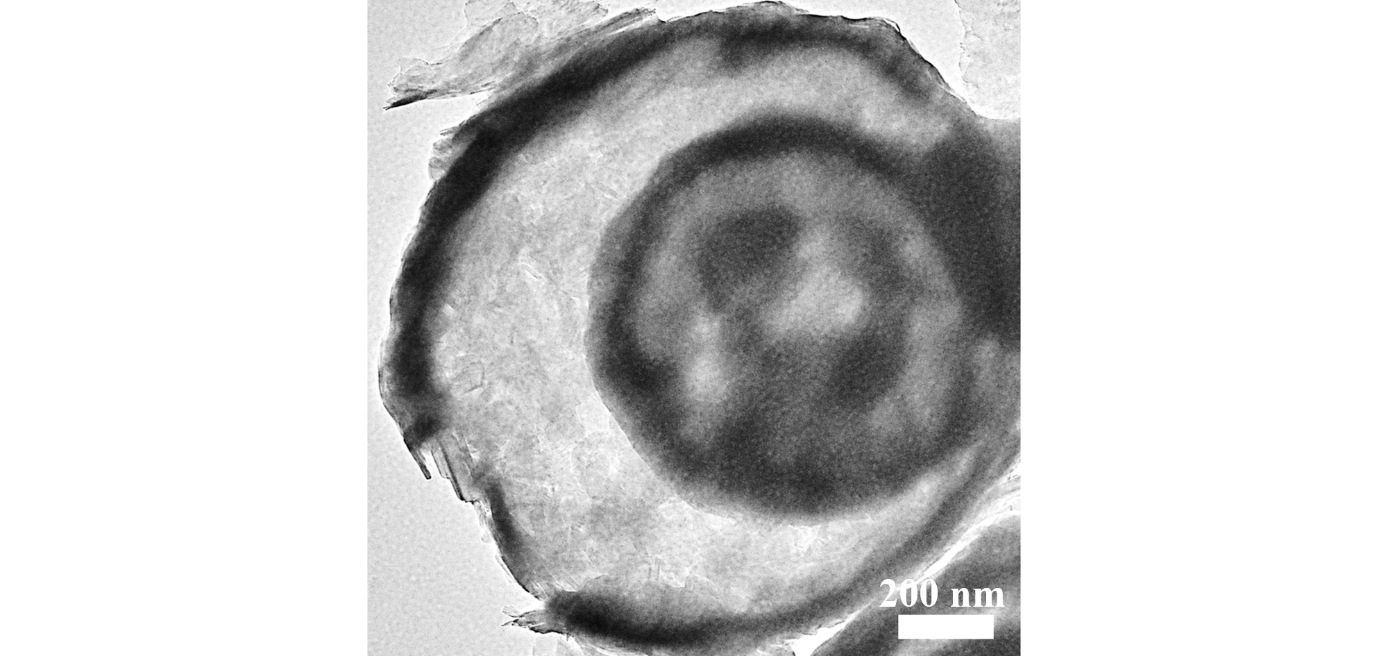


**Figure S11.** TEM image of FeS/MoS_2_ hollow sphere.


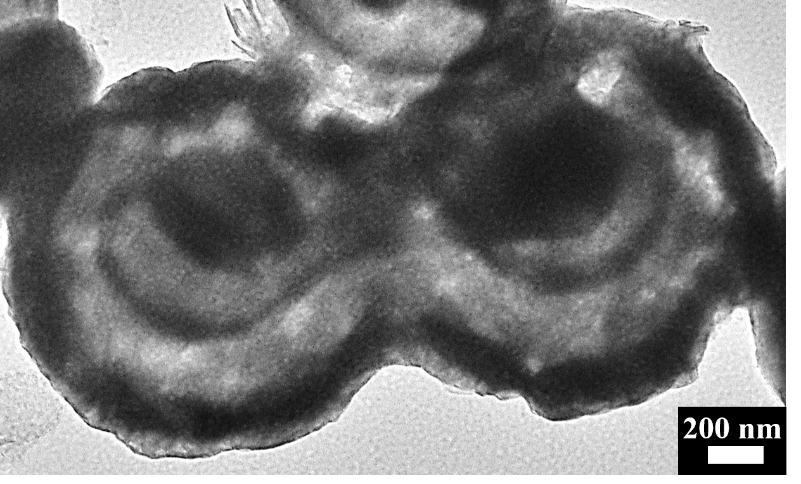


**Figure S12.** TEM image of T-FeS/MoS_2_@NC.


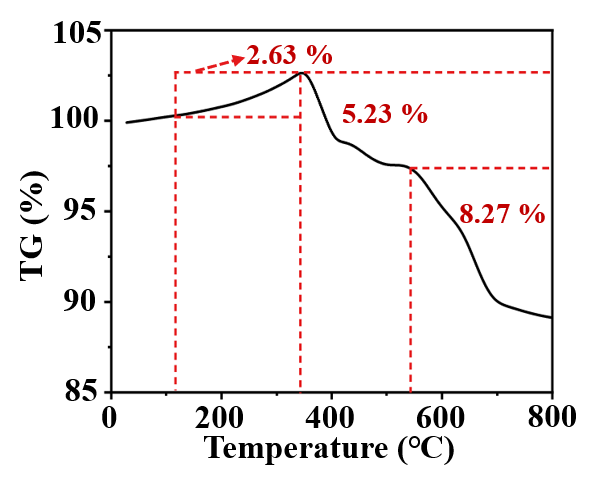


**Figure S13.** TGA curves of FeS/MoS_2_@NC nanocomposite in air with a heating rate of 10 °C min^–1^ from room temperature to 800 °C.


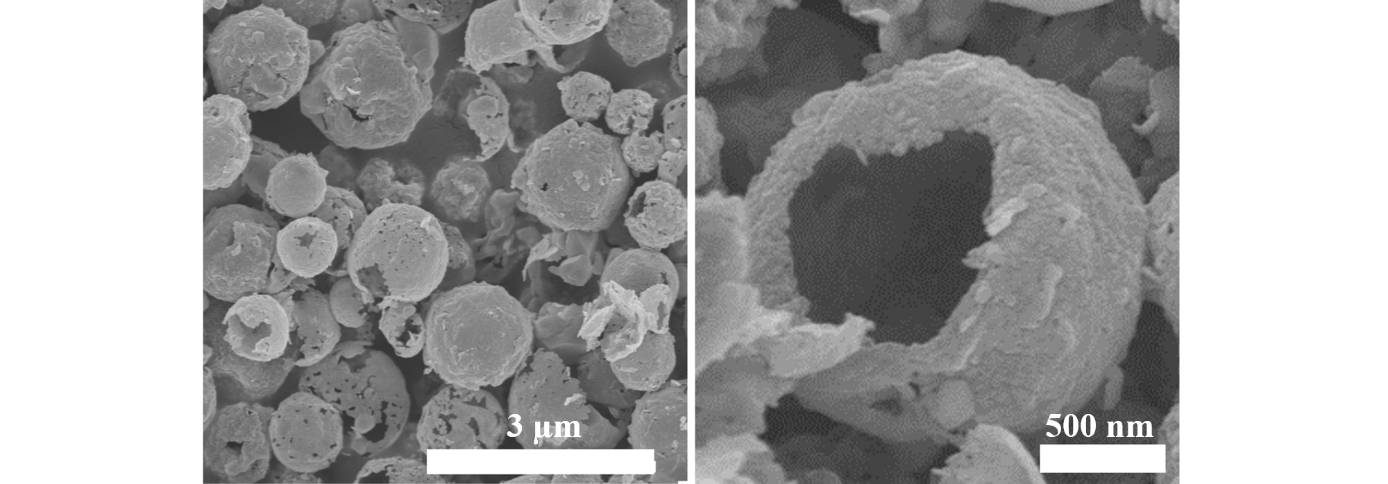


**Figure S14.** SEM images of S-FeS/MoS_2_@NC at different magnifications.


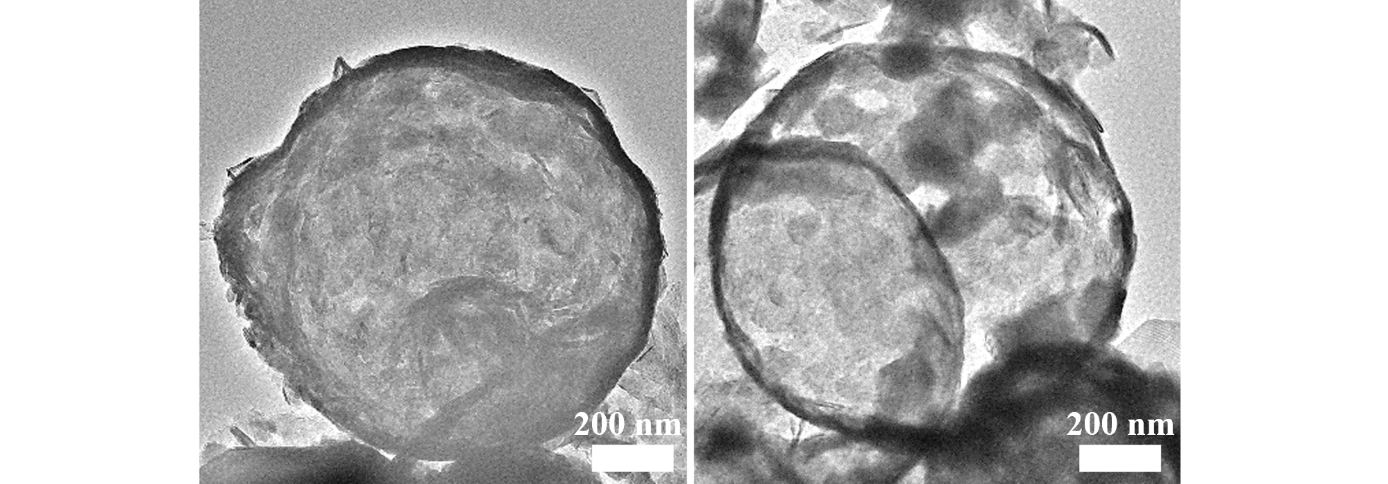


**Figure S15.** TEM images of S-FeS/MoS_2_@NC at different magnifications.


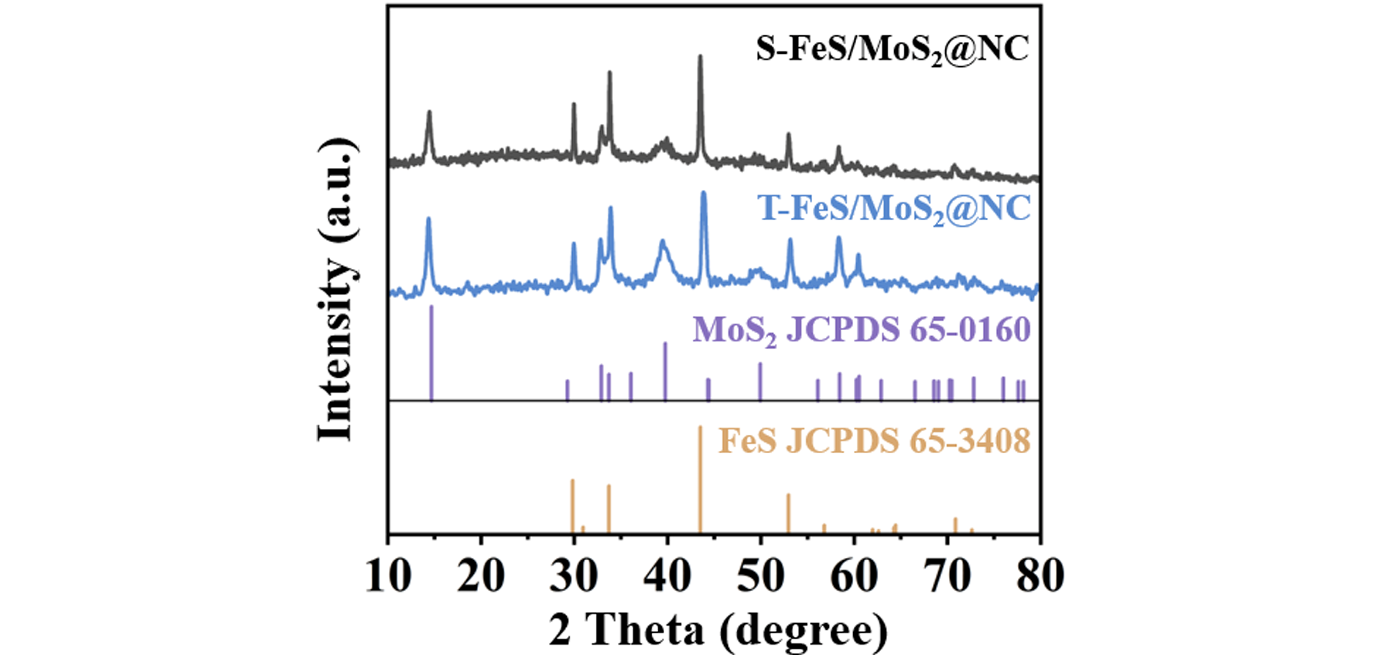


**Figure S16**. XRD pattern of S-FeS/MoS_2_@NC and T-FeS/MoS_2_@NC.


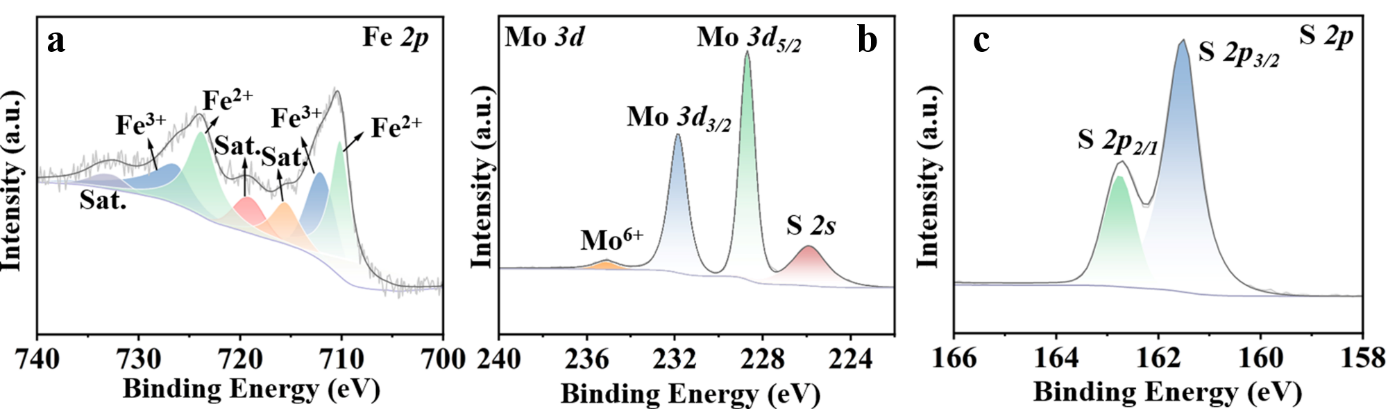


**Figure S17**. High-resolution XPS spectra of T-FeS/MoS_2_@NC: (a) Fe 2*p*, (b) Mo 3*d* and (c) S 2*p*.


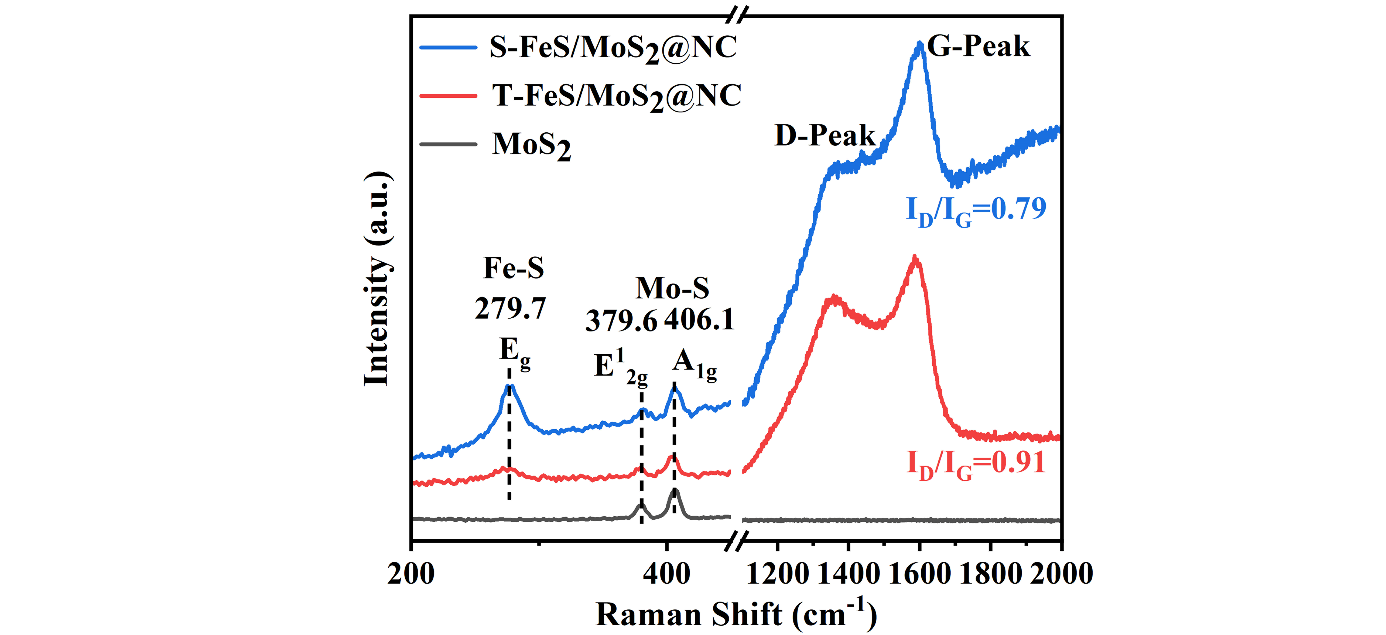


**Figure S18**. Raman spectra of T-FeS/MoS_2_@NC, S-FeS/MoS_2_@NC and MoS_2_.


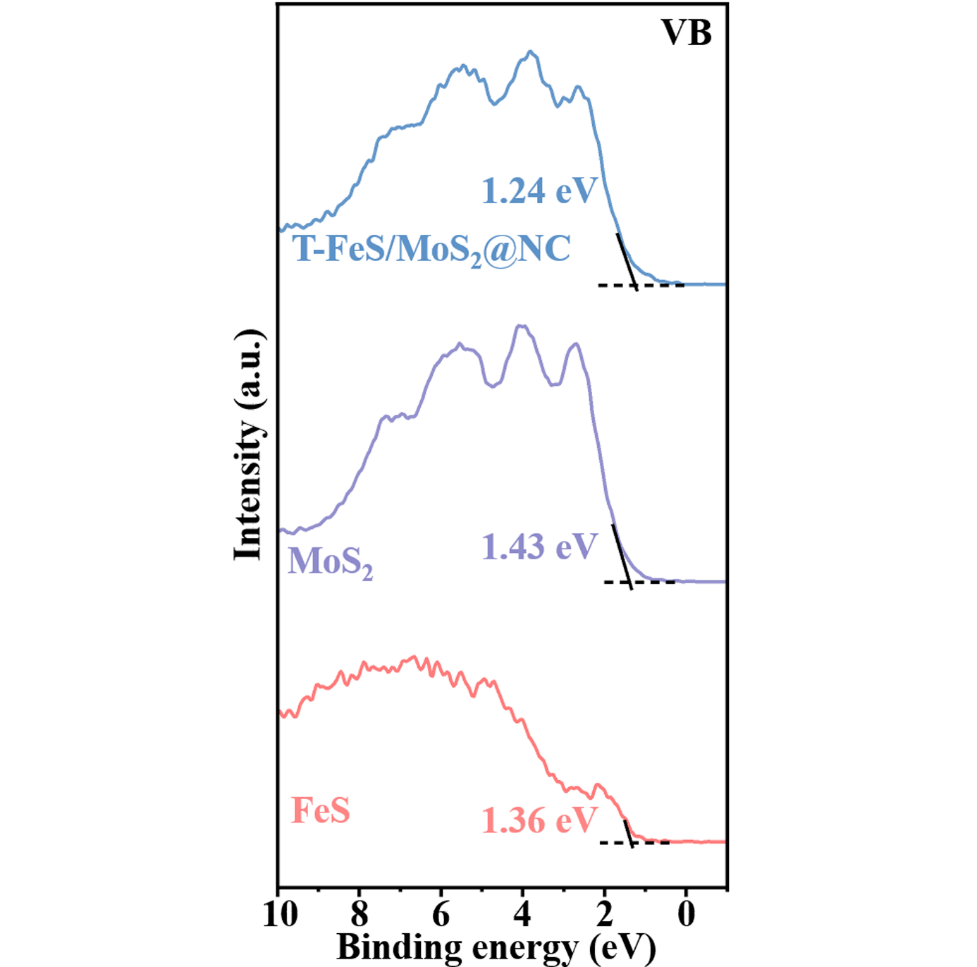


**Figure S19.** VB-XPS spectra of T-FeS/MoS_2_@NC, MoS_2_ and FeS.


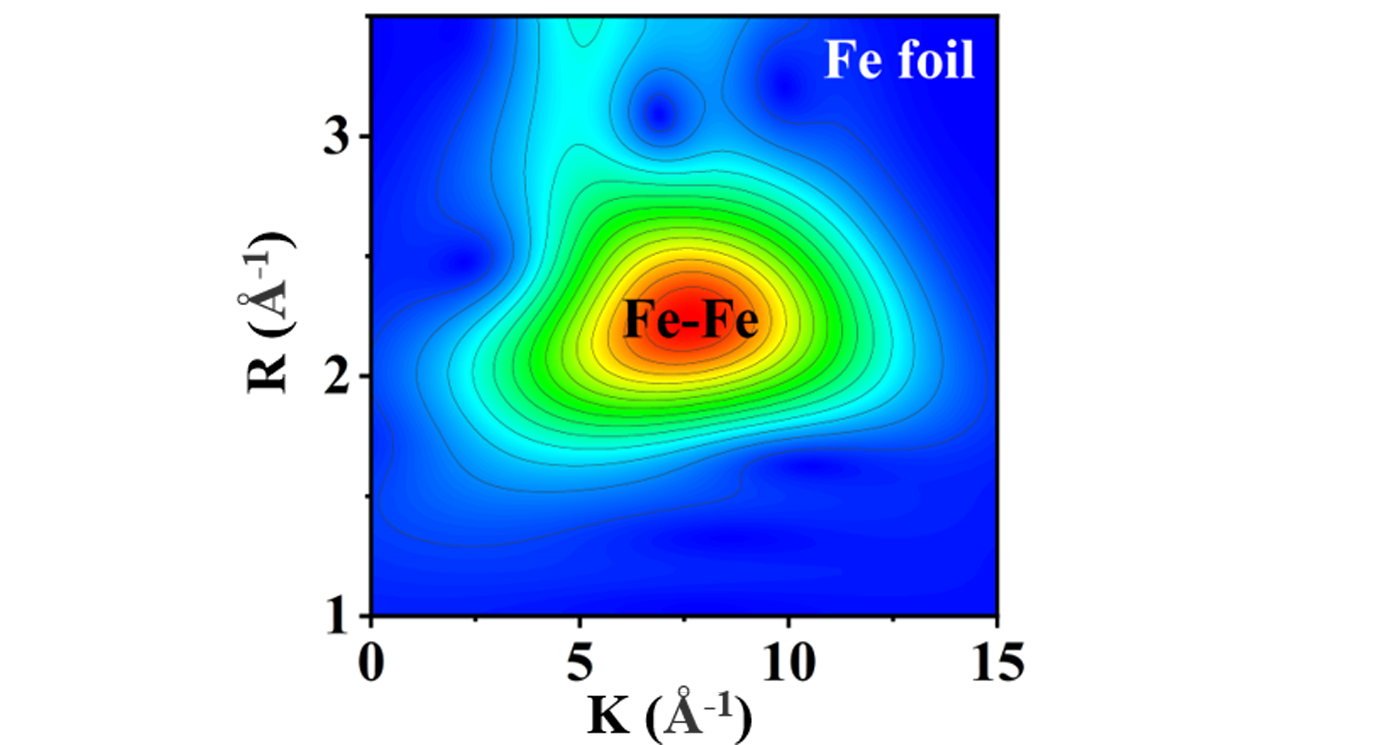


**Figure S20.** Wavelet transform diagram of Fe foil.


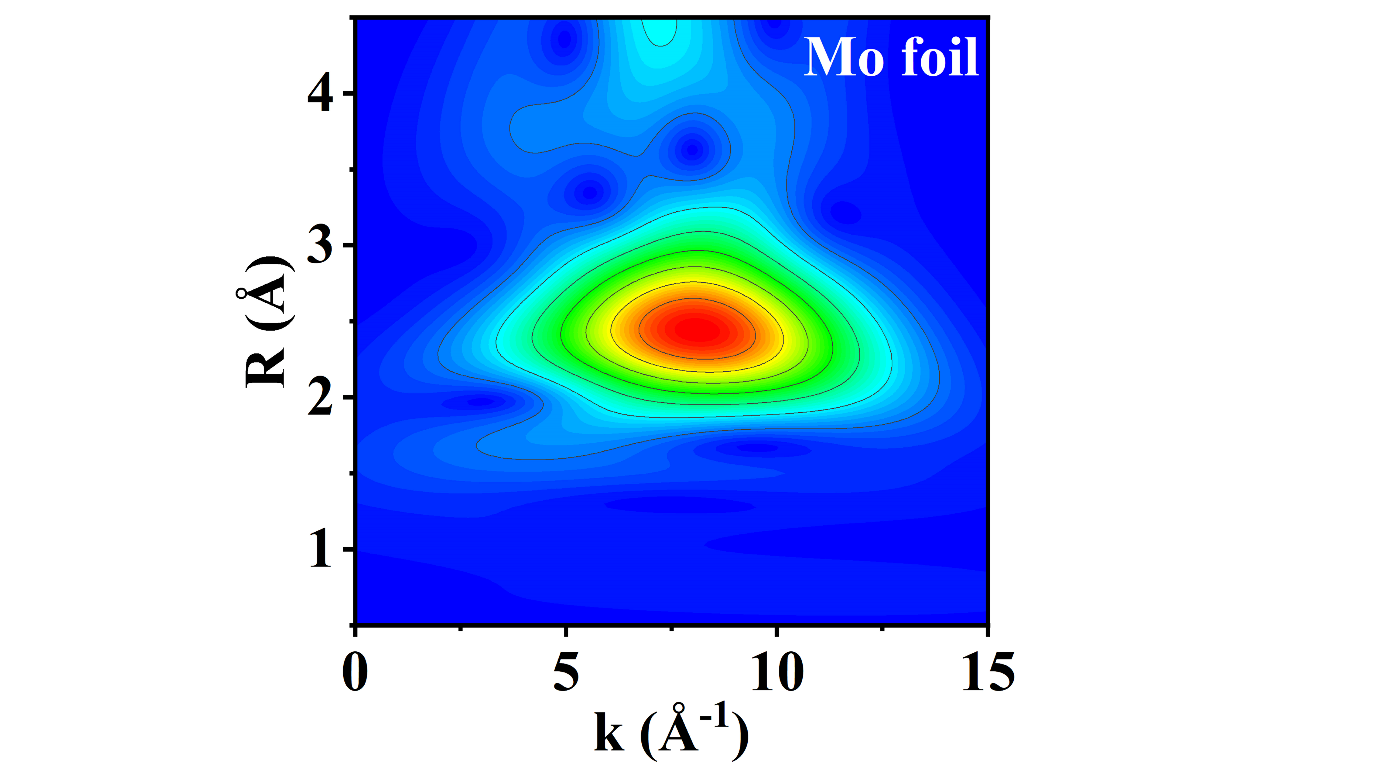


**Figure S21.** Wavelet transform diagram of Mo foil.


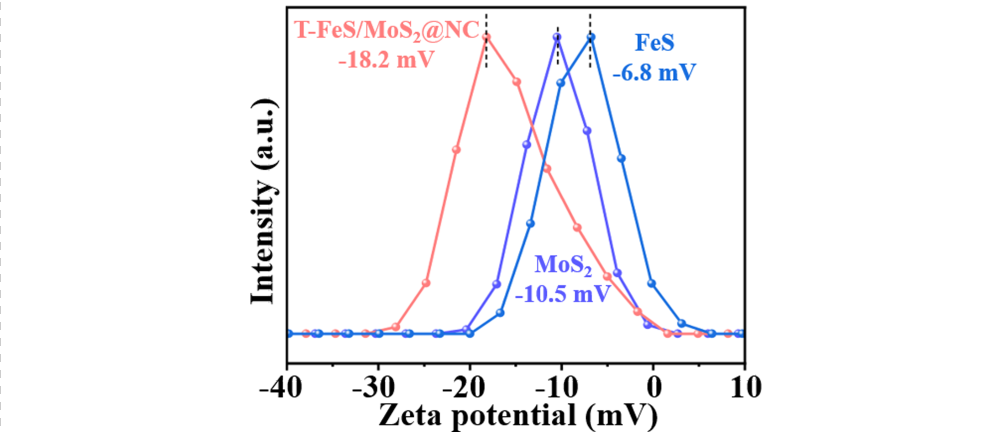


**Figure S22**. Zeta potentials of T-FeS/MoS_2_@NC, MoS_2_ and FeS.


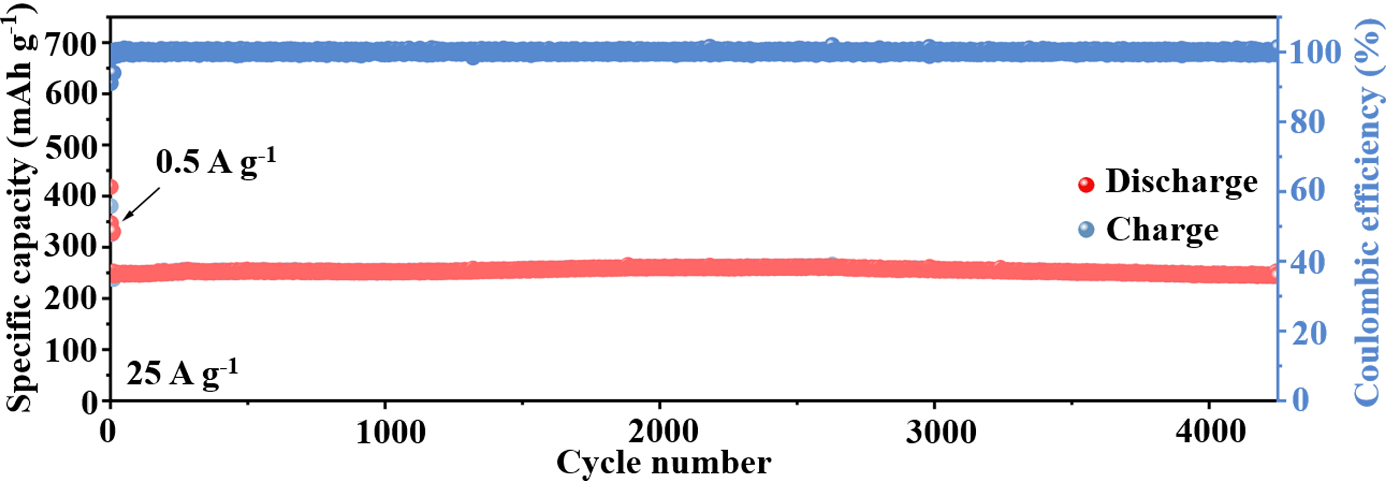


**Figure S23.** The cycling performance of the T-FeS/MoS_2_@NC electrode at a current density of 25 A g^–1^.


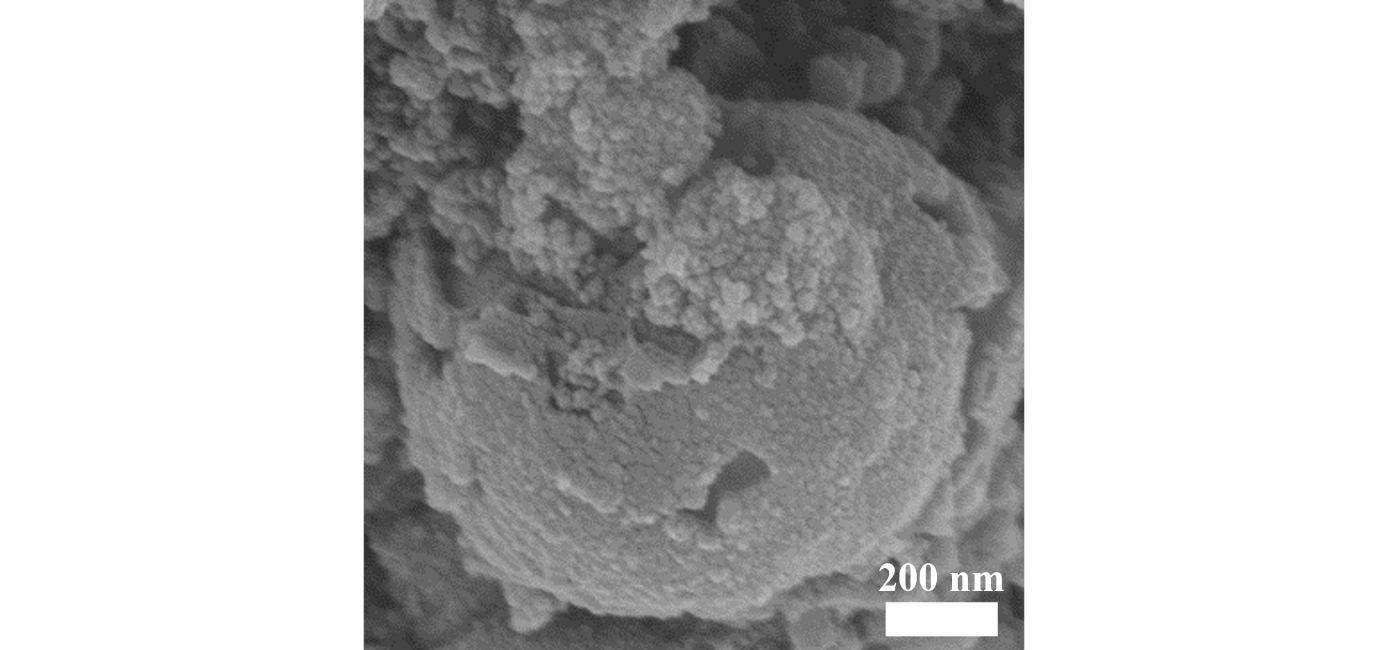


**Figure S24.** The SEM image of T-FeS/MoS_2_@NC after 100 cycles at 0.5 A g^–1^_._


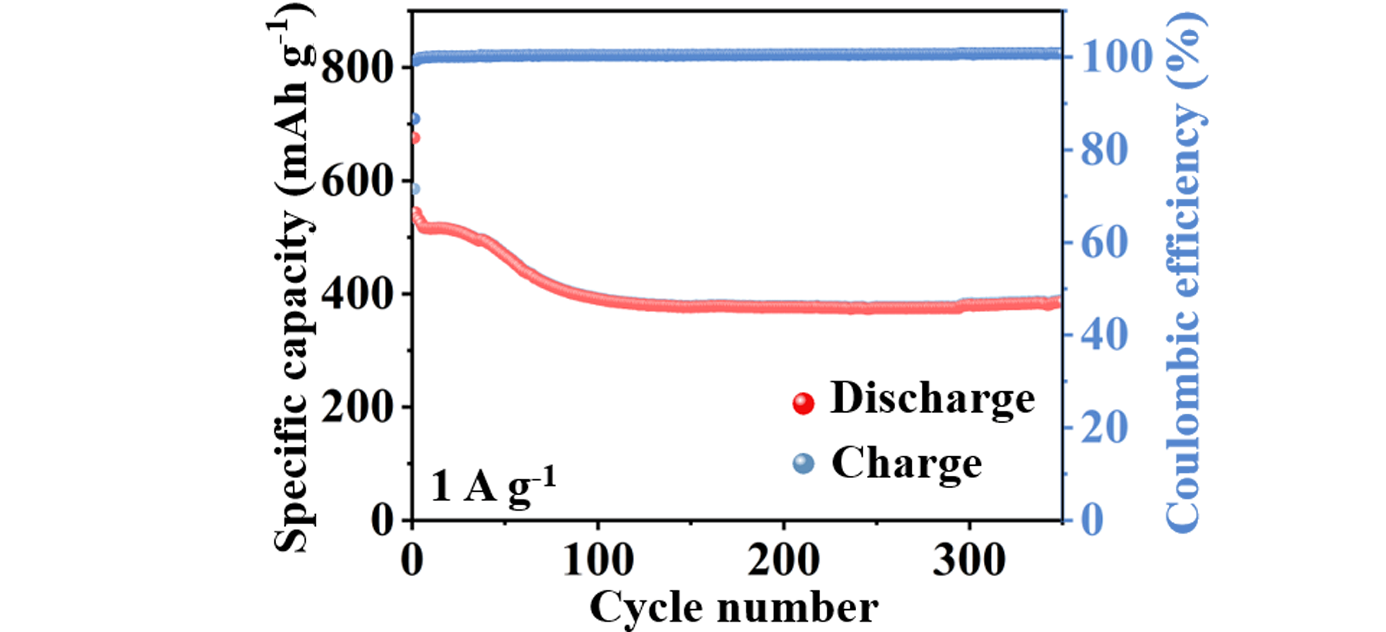


**Figure S25.** Cycling performance of FeS/MoS_2_ electrode material at 1 A g^–1^.


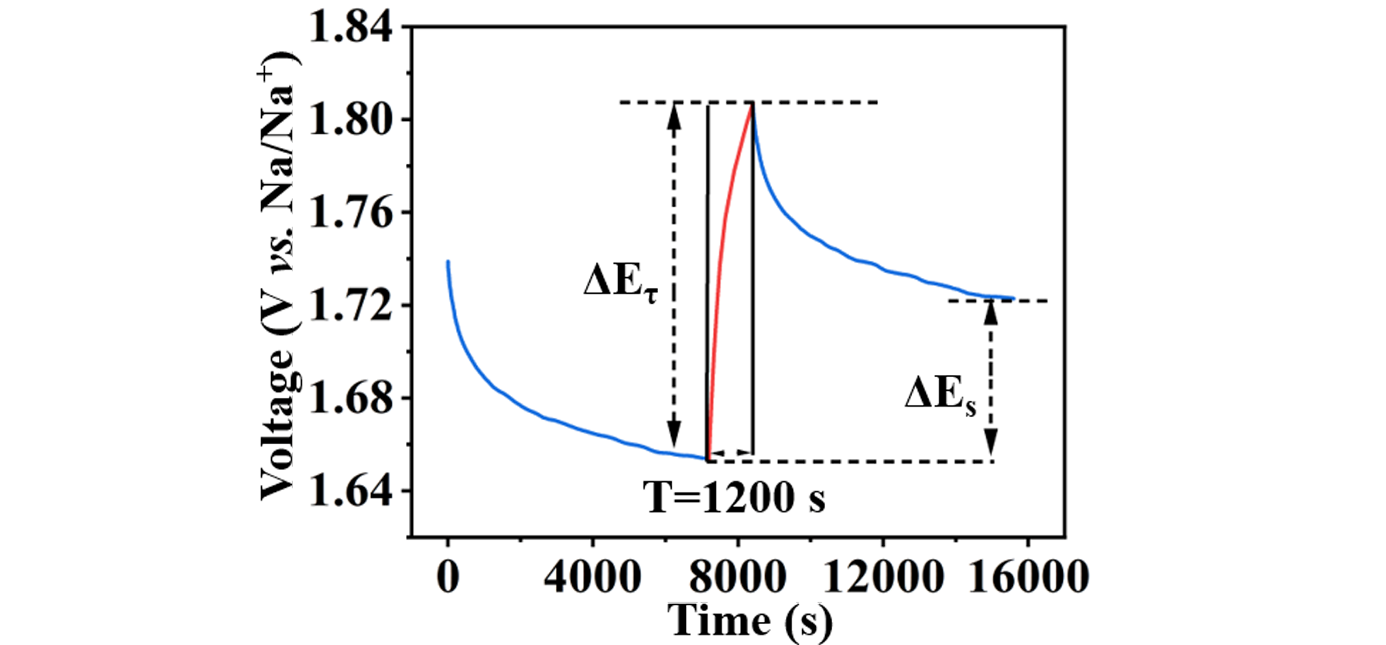


**Figure S26.** The detailed GITT diagram with a single current pulse for 1200 s followed by an open-circuit for 7200 s.


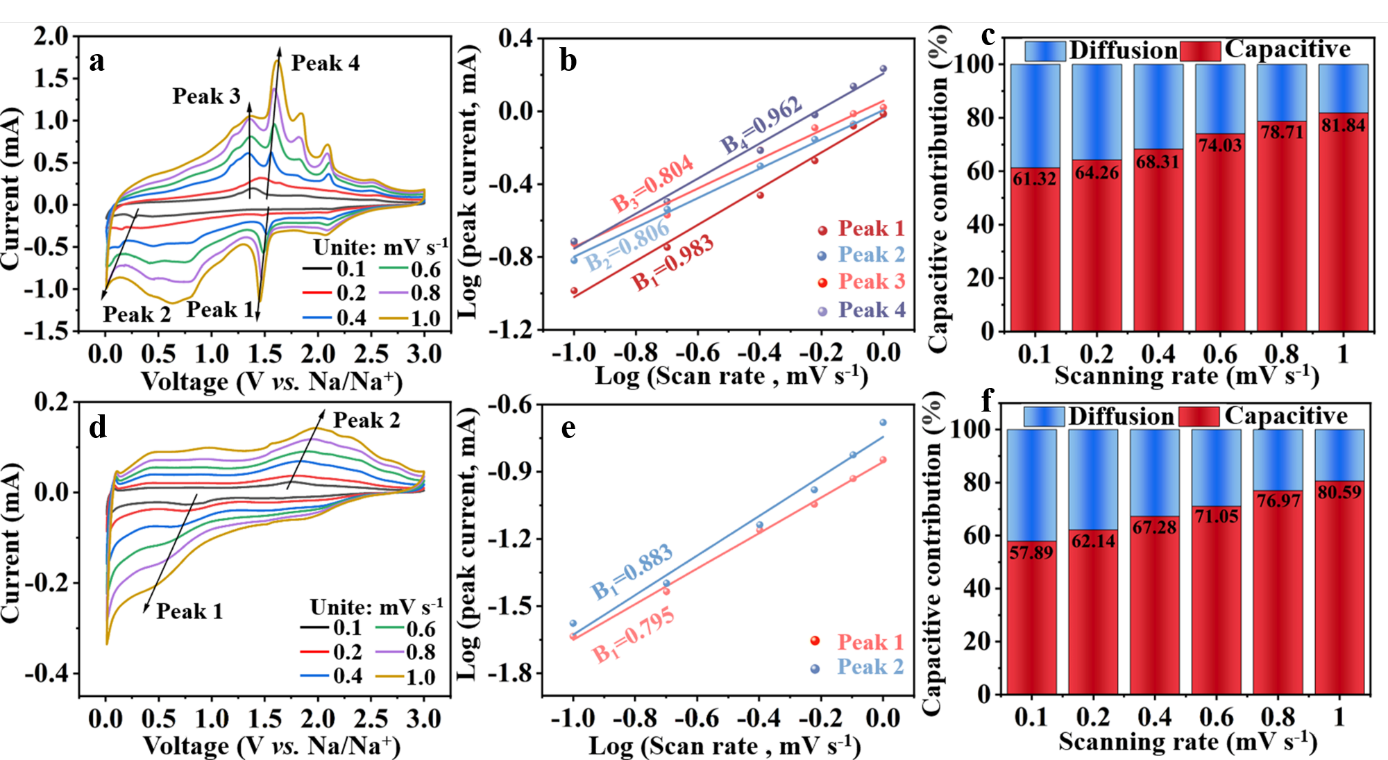


**Figure S27**. (a) CV curves, (b) corresponding log(*i*) *vs* log(*v*) plots, and (c) pseudocapacitive contributions of S-FeS/MoS_2_@NC at different scan rates. (d) CV curves, (e) corresponding log(*i*) *vs* log(*v*) plots, and (f) pseudocapacitive contributions of MoS_2_ at different scan rates.


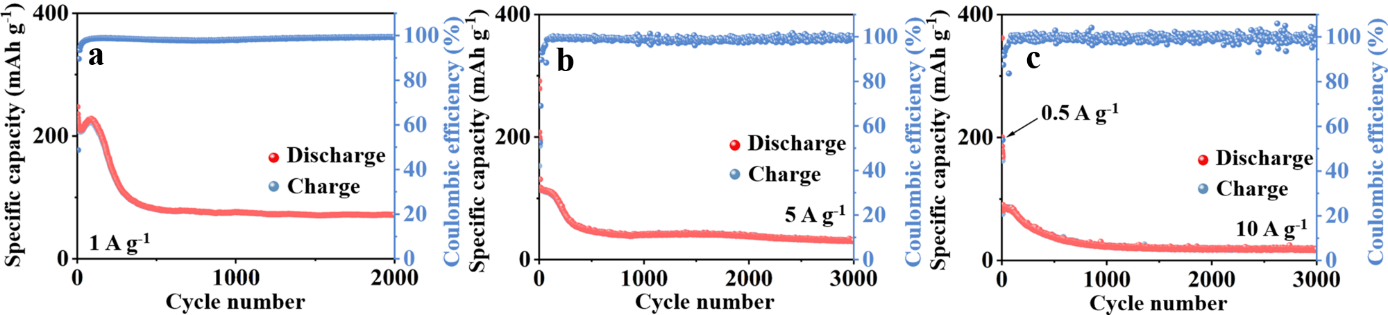


**Figure S28.** The cycling performances of the T-FeS/MoS_2_@NC electrode material were evaluated using a carbonate-based electrolyte consisting of 1 M NaClO_4_ in a mixture of ethylene carbonate (EC), diethyl carbonate (DEC), and ethyl methyl carbonate (EMC) (1:1:1, v/v/v). The tests were conducted at current densities of (a) 1 A g^–1^, (b) 5 A g^–1^, and (c) 10 A g^–1^.


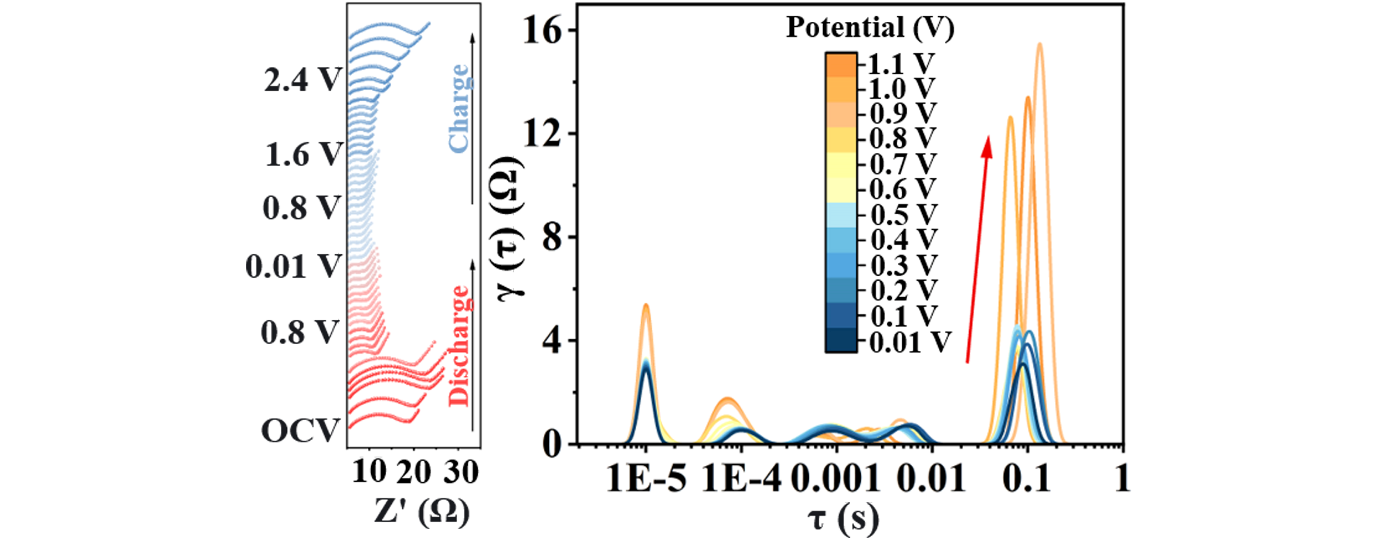


**Figure S29**. The *in-situ* impedances of the S-FeS/MoS_2_@NC electrode in the first cycle and the DRT fitting results from 1.1 V to 0.01 V.


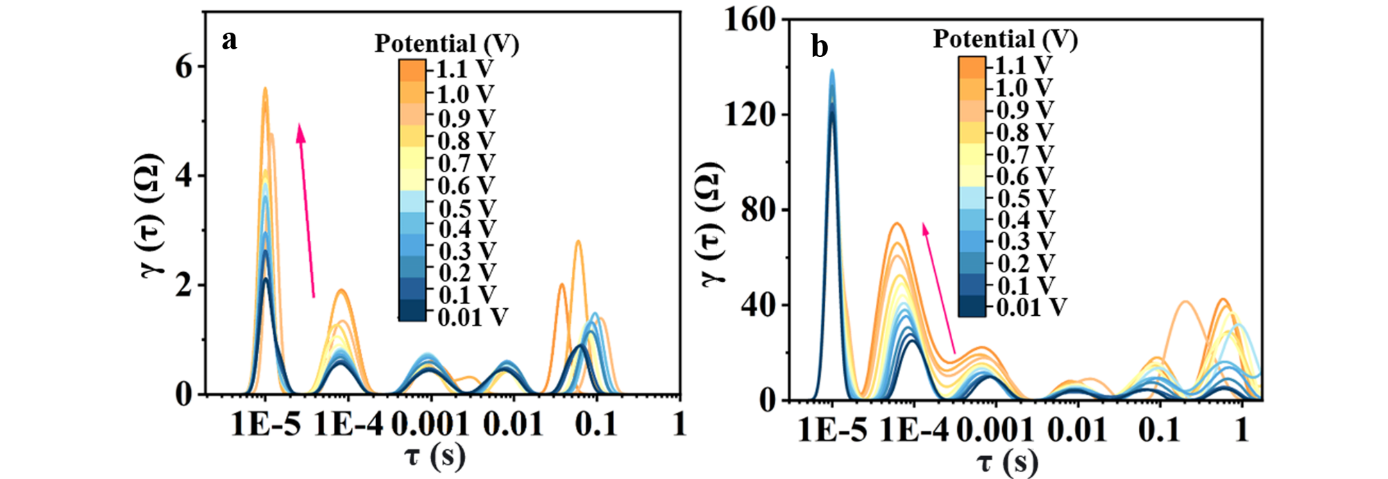


**Figure S30.** DRT fitting results of (a) T-FeS/MoS₂@NC and (b) MoS₂ electrodes in the first cycle with a voltage range from 1.1 V to 0.01 V.


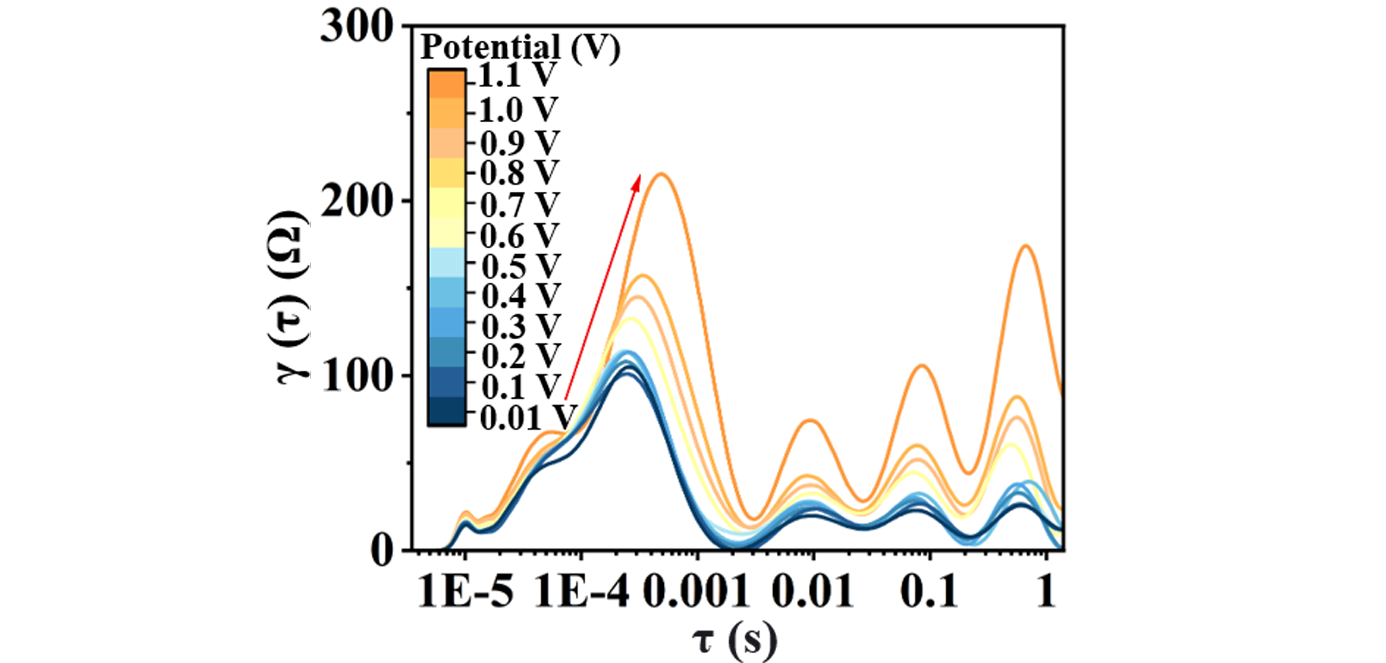


**Figure S31.** The in-situ impedance of the T-FeS/MoS_2_@NC electrode was measured using a carbonate-based electrolyte consisting of 1M NaClO_4_ in a 1:1:1 (v/v/v) mixture of ethylene carbonate (EC), diethyl carbonate (DEC), and ethyl methyl carbonate (EMC). The DRT fitting results were obtained over a voltage range of 1.1 V to 0.01 V.


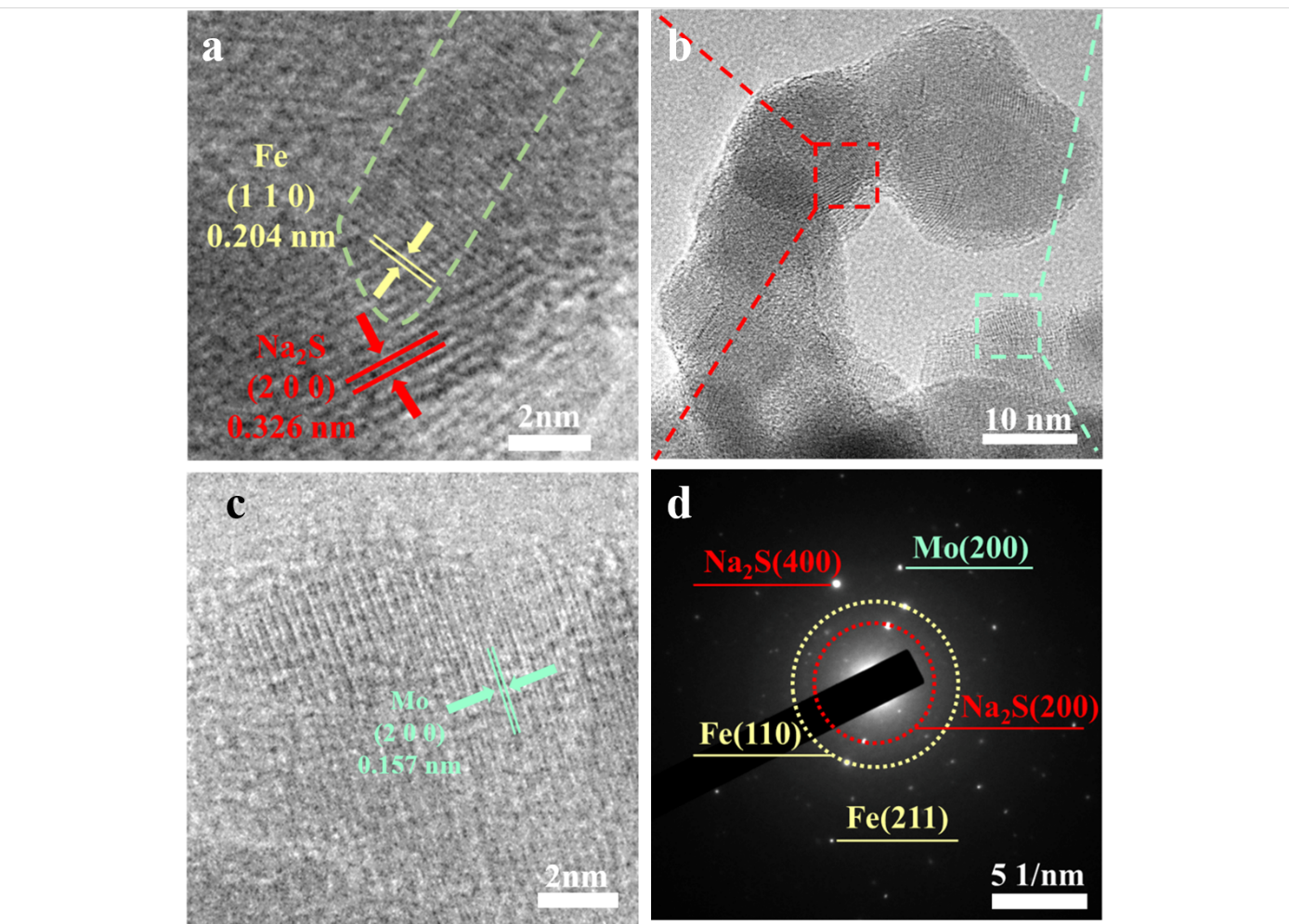


**Figure S32**. (a-c) The *ex-situ* HRTEM images and (d) the corresponding SAED pattern when T-FeS/MoS_2_@NC is discharged to 0.01V.


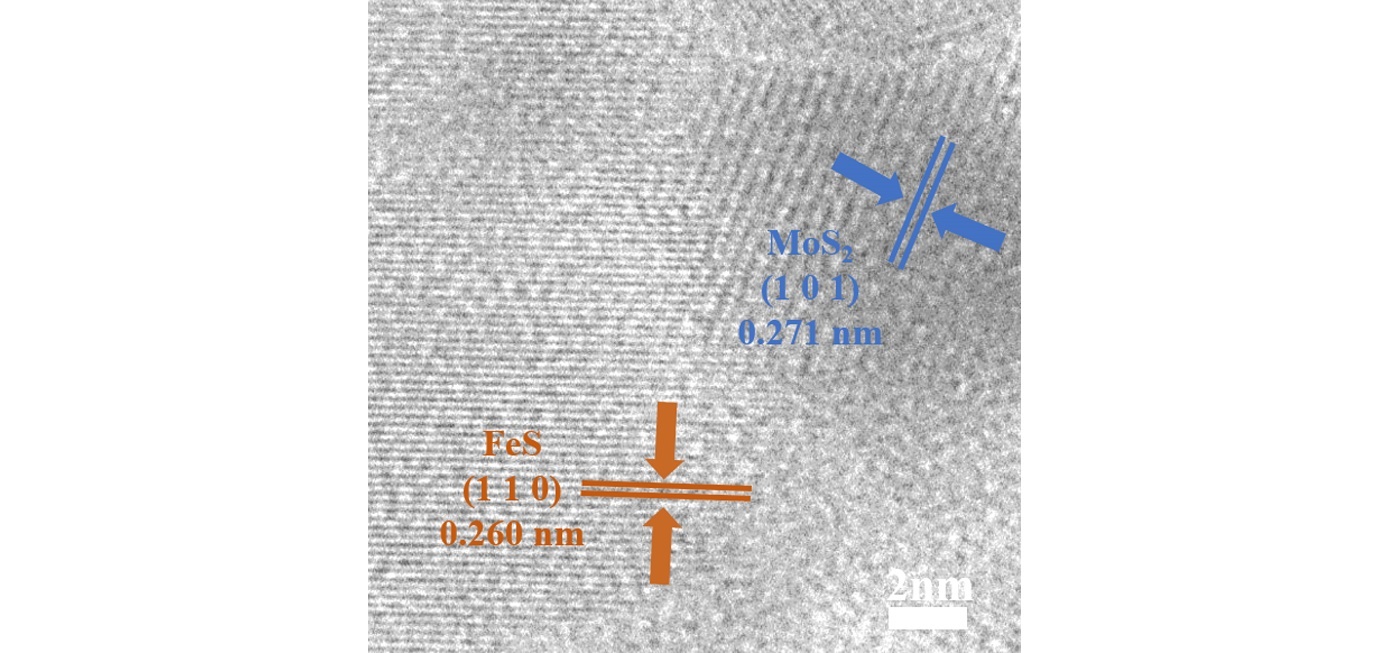


**Figure S33.** *Ex-situ* HRTEM image of T-FeS/MoS_2_@NC anode after being fully charged to 3.0 V.


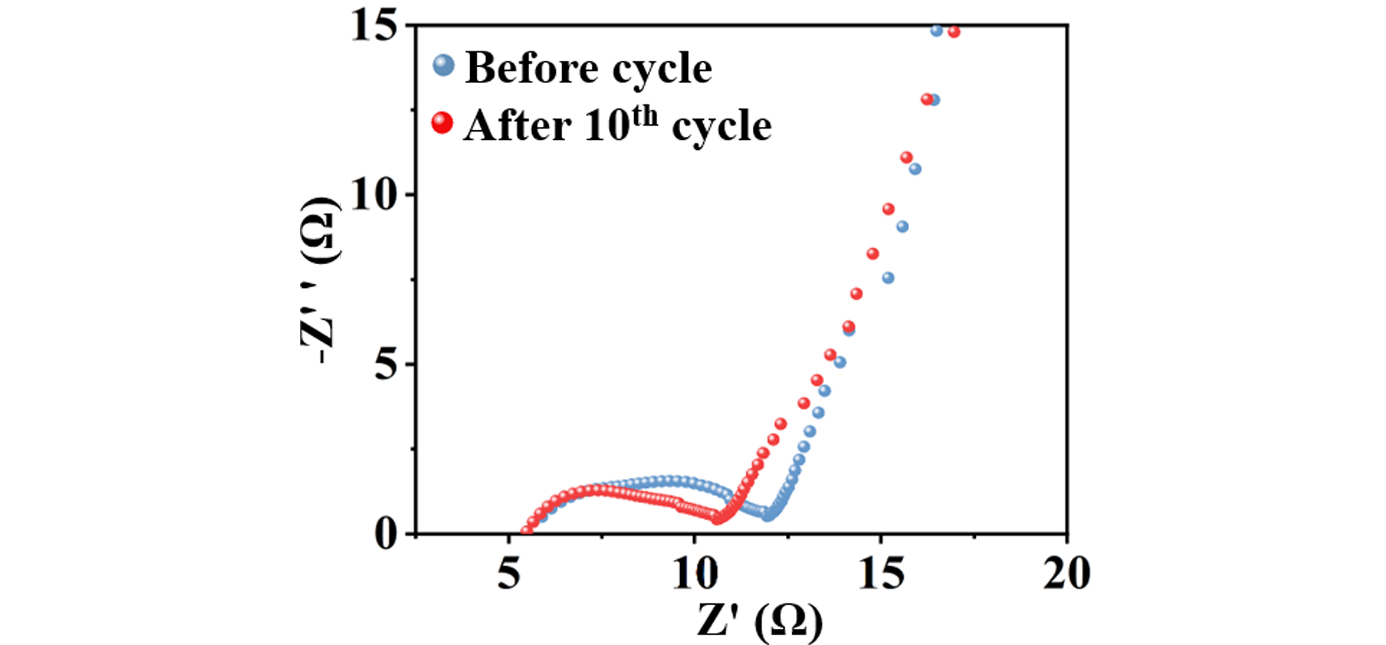


**Figure S34**. EIS images of T-FeS/MoS_2_@NC before and after 10 cycles at a current density of 1 A g^–1^.


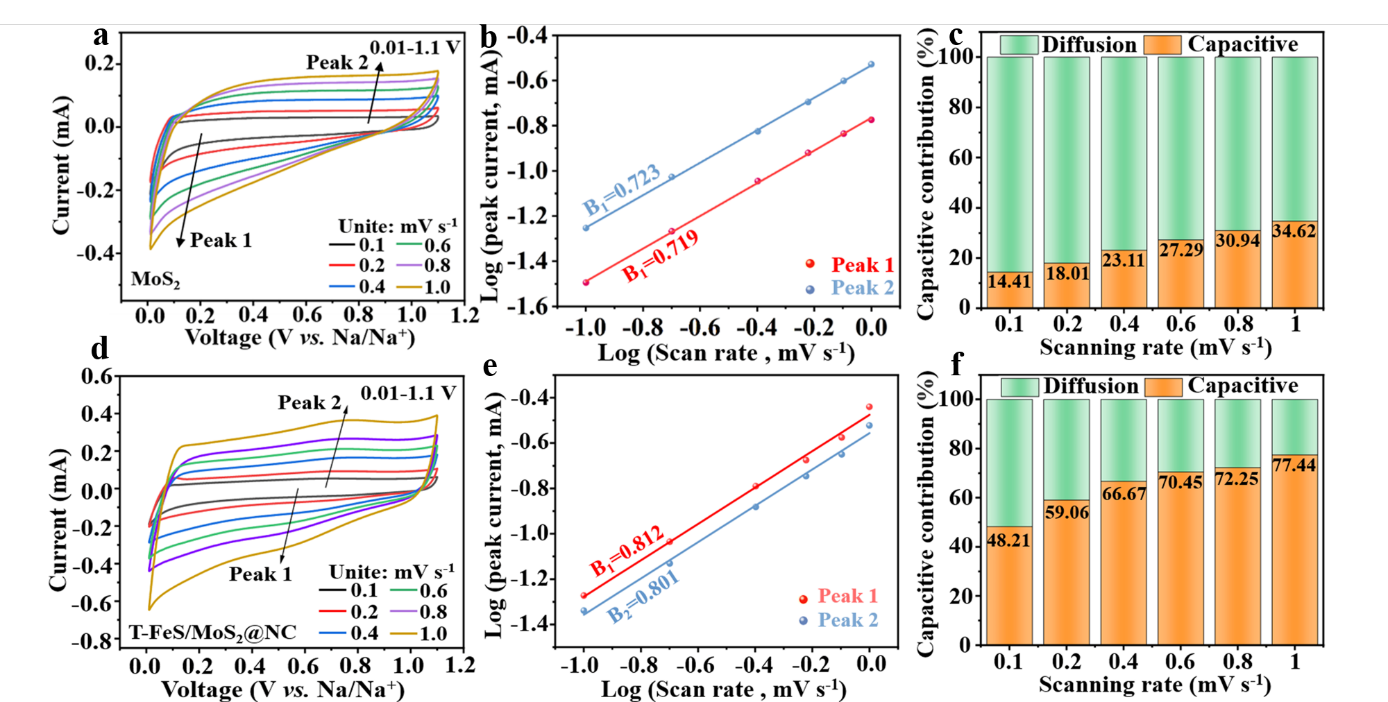


**Figure S35**. CV curves of (a) T-FeS/MoS_2_@NC, and (d) MoS_2_ at different scan rates within a voltage window of 0.01~1.1 V. The corresponding log (*i*) vs log (*v*) plots of (b) T-FeS/MoS_2_@NC and (e) MoS_2_. Pseudo-capacitive contributions of (c) T-FeS/MoS_2_@NC and (f) MoS_2_ at different scan rates within a voltage window of 0.01~1.1 V.


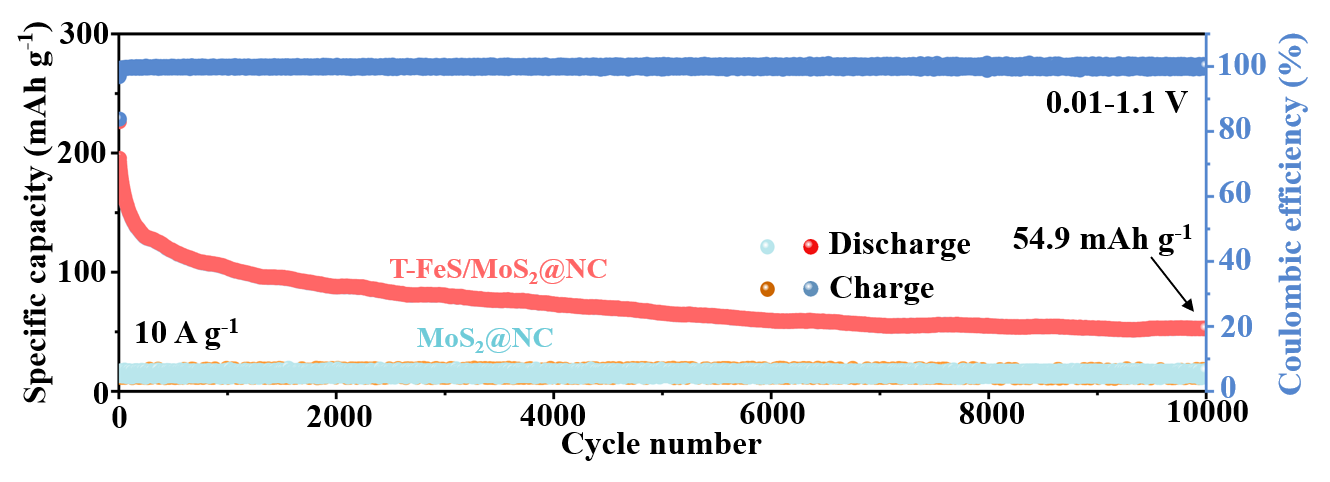


**Figure S36.** Cycling performances of T-FeS/MoS_2_@NC and MoS_2_@NC electrodes at a current density of 10 A g^–1^ within a voltage window of 0.01~1.1 V.


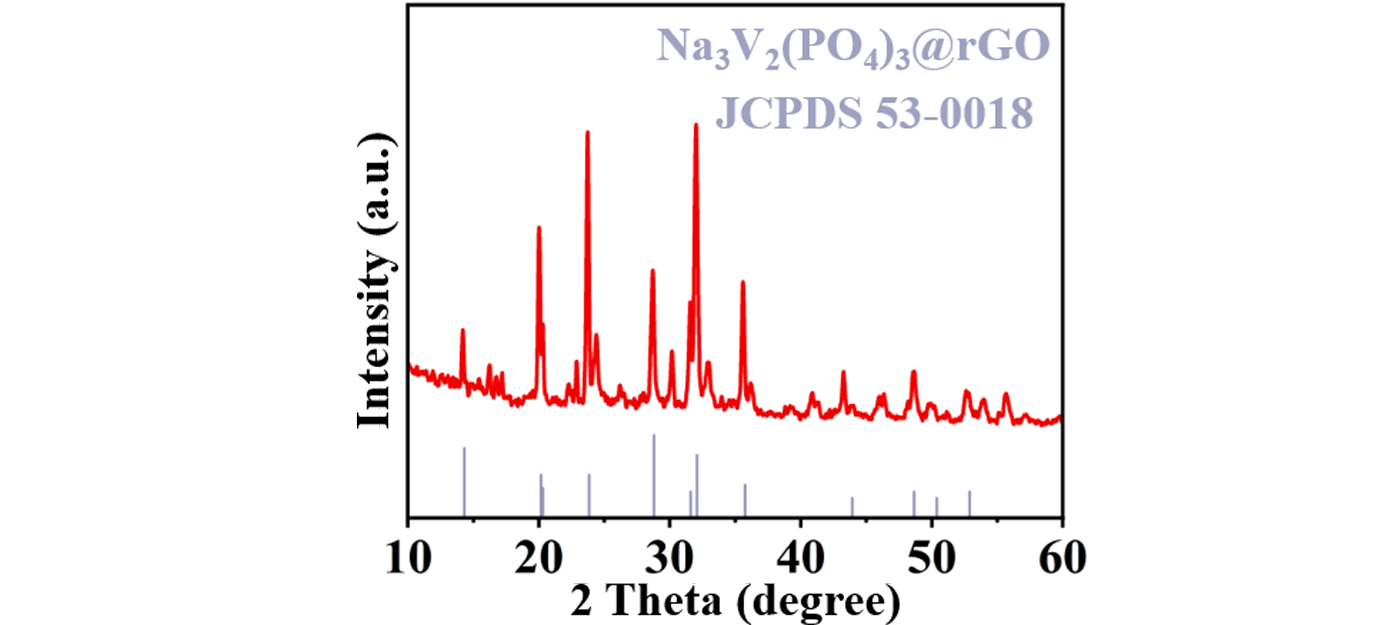


**Figure S37**. A typical XRD pattern of NVP@rGO.

**Table S1.** The inductively coupled plasma optical emission spectroscopy (ICP-OES) analysis results of FeS/MoS_2_@NC.

| **Elements** | **Content (mg/L)** | **Content (mol/L)** | **Mole Ration**  **(Fe : Mo)** |
| --- | --- | --- | --- |
| Fe | 2.404 | 0.043 | 5 : 7 |
| Mo | 5.835 | 0.060 |  |

**Table 2.** Comparison of cycling stability and specific capacity between T-FeS/MoS_2_@NC and previously reported metal sulfide anodes for SIBs.

| Materials | Current density  [A g^–1^] | Cycles | Specific capacity  [mAh g^–1^] | Refs. |
| --- | --- | --- | --- | --- |
| T-FeS/MoS_2_@NC | 2.0  5.0 | 700  9000 | 547.6  451.5 | This work |
| MoS_2_@rGO | 2.0 | 600 | 408 | 3 |
| Fe_2_Mo_3_O_8_@C@MoS_2_ | 2.0 | 400 | ~375 | 4 |
| MoS_2_/VS_2_ | 2.0 | 800 | 451.6 | 5 |
| Co-MoS_2_@NC | 5.0 | 8240 | 218.6 | 6 |
| FeS@FeC_3_/GC | 5.0 | 200 | ~130 | 7 |
| MoS_2_@NiS_1.03_-C | 5.0 | 1000 | 313.5 | 8 |
| MoS_2_-SnS@g-C_3_N_4_/G | 2.0 | 500 | 320 | 9 |
| MoS_x_@HPC | 2.0 | 500 | 484 | 10 |
| MoO_3_-MoS_2_ | 5.0 | 2300 | 286 | 11 |
| MoS_2_/SnS@C | 5.0 | 2000 | 289 | 12 |
| MoS_2_ Nss@TiO_2_ NFs | 5.0 | 1100 | 298.4 | 13 |

**Table 3.** Comparison of rate capacity between T-FeS/MoS_2_@NC and previously reported metal sulfide anodes for SIBs.

| Materials | Rate capacity/current  density  [mAh g^–1^/A g^–1^] | Refs. |
| --- | --- | --- |
| T-FeS/MoS_2_@NC | 590.1/0.1  538.1/0.2  468.9/0.5  419.6/1.0  385/2.0  329.3/5.0 | This work |
| MoS_2_@NHCS | 494/0.1  475/0.2  425/0.5  377/1.0  313/2.0  204/5.0 | 14 |
| Co-MoS_2_(1:8)/3DNC | 438/0.1  414/0.2  387/0.5  363/1.0  350/2.0  327/5.0 | 15 |
| Cu_2_S@carbon@MoS_2_ | 410/0.1  386/0.2  359/0.5  337/1.0  316/2.0 | 16 |
| P-MoS_2_@C/CNTP | 540/0.2  454/0.5  394/1.0  294/2.0  191/5.0 | 17 |
| Fe_3_O_4_/Fe/FeS | 375.6/0.1  329.4/0.5  297.8/1.0  218.8/2.0  149.5/5.0 | 18 |

**References**

[1] S. Clark, M. Segall, C. Pickard, P. Hasnip, M. Probert, K. Refson and M. Payne, *Z. Kristallogr.* **2005**, *220*, 567.

[2] J. Perdew, K. Burke and M. Ernzerhof, *Phys. Rev. Lett.* **1996**, *77*, 3865.

[3] L. Jing, J. Sun, C. Sun, D. Wu, G. Lian, D. Cui, Q. Wang, H. Yu, *Nano Res.* **2022**, *16*, 473.

[4] Y. Tang, G. Li, S. Cui, W. Cui, H. Chong, L. Han, H. Pang, *Adv. Funct. Mater.* **2024**, *34*, 2403351.

[5] X. Yue, J. Wang, A. M. Patil, X. An, Z. Xie, X. Hao, Z. Jiang, A. Abudula, G. Guan, *Chem. Eng. J.* **2021**, *417*, 128107.

[6] P. Li, Y. Yang, S. Gong, F. Lv, W. Wang, Y. Li, M. Luo, Y. Xing, Q. Wang, S. Guo, *Nano Res.* **2018**, *12*, 2218.

[7] Q. Wang, W. Zhang, C. Guo, Y. Liu, C. Wang, Z. Guo, *Adv. Funct. Mater.* **2017**, *27*, 1703390.

[8] S. Gao, Y. He, G. Yue, H. Li, S. Li, J. Liu, B. Miao, J. Bai, Z. Cui, N. Wang, Q. Zhang, L. Jiang, Y. Zhao, *Carbon Energy* **2023**, *5*, 319.

[9] X. Yuan, S. Qiu, X. Zhao, *ACS Appl. Mater. Interfaces* **2021**, *13*, 34238.

[10] J. Rahmatinejad, X. Liu, X. Zhang, B. Raisi, Z. Ye, J. *Energy Chem.* **2022**, *75*, 240.

[11] L. Yu, X. Tao, D. Sun, L. Zhang, C. Wei, L. Han, Z. Sun, Q. Zhao, H. Jin, G. Zhu, *Adv. Funct. Mater.* **2024**, *34*, 2311471.

[12] L. Tang, B. Zhang, T. Peng, Z. He, C. Yan, J. Mao, K. Dai, X. Wu, J. Zheng, *Nano Energy* **2021**, *90*, 106568.

[13] K. Zhu, S. Gao, T. Bai, H. Li, X. Zhang, Y. Mu, W. Guo, Z. Cui, N. Wang, Y. Zhao, *Small* **2024**, *20*, 2402774.

[14] M. Hou, Y. Qiu, G. Yan, J. Wang, D. Zhan, X. Liu, J. Gao, L. Lai, *Nano Energy* **2019**, *62*, 299.

[15] H. Xie, B. Chen, C. Liu, G. Wu, S. Sui, E. Liu, G. Zhou, C. He, W. Hu, N. Zhao, *Energy Stor. Mater.* **2023**, *60*, 102830.

[16] Y. Fang, D. Luan, Y. Chen, S. Gao, X. W. D. Lou, *Angew. Chem. Int. Ed.* **2020**, *5*9, 7178.

[17] S. Sui, H. Xie, M. Liang, B. Chen, C. Liu, E. Liu, B. Chen, L. Ma, J. Sha, N. Zhao, *Adv. Funct. Mater.* **2022**, *32*, 2110853.

[18] Y. Liu, Q. Lin, X. Chen, X. Meng, B. Hou, H. Liu, S. Zhang, N. Shang, Z. Wang, C. Zhang, J. Song, X. Zhao, *Energy Environ. Mater.* **2023**, *7*, 12684.
